# A dual respiratory and auditory function for the coelacanth lung

**DOI:** 10.1038/s42003-026-09708-6

**Published:** 2026-02-14

**Authors:** Luigi Manuelli, Gaël Clément, Marc Herbin, Bernd Fritzsch, Per E. Ahlberg, Kathleen Dollman, Lionel Cavin

**Affiliations:** 1 Department of Earth Sciences, Natural History Museum of Geneva, Geneva, Switzerland; 2 Department of Genetics and Evolution, University of Geneva, Geneva, Switzerland; 3 Centre de Recherche en Paléontologie-Paris (CR2P, MNHN-CNRS-Sorbonne Université), Muséum National d’Histoire Naturelle, Paris, France; 4 Mécanismes Adaptatifs et Evolution (MECADEV, MNHN-CNRS-Sorbonne Université) Muséum National d’Histoire Naturelle, Paris, France; 5 Department of Neuronal Sciences, University of Nebraska Medical Center, Omaha, NE USA; 6 Department of Organismal Biology, Uppsala University, Uppsala, Sweden; 7 European Synchrotron and Radiation Facility, Grenoble, France

**Keywords:** Palaeontology, Evolutionary developmental biology

## Abstract

Since the discovery of *Latimeria chalumnae*, coelacanths have provided a critical comparative framework for reconstructing ancestral sarcopterygian anatomy. However, the function of several anatomical features in both extant and fossil coelacanths remains unresolved. Among these, the presence of large ossified chambers in the body cavity of fossil coelacanths has remained enigmatic, with different studies proposing respiratory or auditory functions. Here, we examine lung and inner ear anatomy based on new observations from synchrotron phase-contrast microCT scans of two 240-million-year-old latimerioid coelacanths, alongside multiple developmental stages of the extant *L. chalumnae*. These data, combined with archival histological sections of *L. chalumnae* and 3D reconstructions of a Devonian coelacanth, suggest that extinct coelacanths possessed an ossified lung capable of transmitting sound pressure to auditory sensory epithelia in the inner ear via a perilymphatic system. We propose that the lung of extinct coelacanths supported both respiratory and auditory functions.

## Abstract (web summary)

Synchrotron imaging of fossil and extant coelacanths reveals that the lung of extinct species likely served both respiratory and auditory functions, transmitting sound pressure to the inner ear via a specialized perilymphatic system

## Introduction

Coelacanths are lobe-finned fishes (sarcopterygians) with a fossil record spanning over 400 million years, representing a key lineage for understanding the evolution of vertebrate anatomy. Once thought extinct, they survive today as the genus *Latimeria*, with two recognized species. Fossil coelacanths exhibit within the body cavity a series of enigmatic large ossified plates arranged in a tile-like pattern, enclosing an internal cavity inferred to have been gas-filled in life^1–3^. In the extant coelacanth *Latimeria chalumnae*, a large gas-filled chamber enclosed by bony plates is absent, although a tiny vestigial lung has been identified^4^. The large ossified gas-filled chamber of fossil coelacanths was recently suggested to have had a respiratory function, hence referred to as an ossified lung, based on coelacanth–tetrapod phylogenetic proximity^1,4–6^, vascular organization^7^, and homology between the large ossified plates in fossil coelacanths and the tiny mineralized plates surrounding the vestigial lung, which includes a compressed lumen, in the extant coelacanth *L. chalumnae*^4,8^. Other functions have also been proposed, including a role in hearing^1^, although no anatomical features had yet been identified to support this hypothesis.

Sound is a mechanical wave composed of sound pressure (variations in pressure) and particle motion (the back-and-forth displacement of molecules). In vertebrates, pressure-sensitive structures such as swim bladders, lungs, or tympanic membranes can detect fluctuations in sound pressure and, in some species, transmit them to the auditory epithelia through fluid-filled perilymphatic pathways. Most bony fishes rely primarily on particle motion detection via the otoliths to perceive sound. However, some taxa can also detect sound pressure through a gas-filled chamber, a diverticulum of the digestive system branching dorsally as a swim bladder or ventrally as lungs. Lungfishes, for instance, can perceive sound transmitted from air within their lungs to the inner ear, despite lacking a specialized lung–ear connection^9^. In several groups of ray-finned fishes, the swim bladder contacts the inner ear either directly or through a chain of ossicles, as in the Weberian apparatus of otophysans (e.g. ref.^10^). This apparatus connects the swim bladder to the inner ear via an unpaired perilymphatic sinus^11^, with the chamber showing varying degrees of ossification across otophysan taxa and, in some cases, being entirely enclosed in bone.

Here, we provide new anatomical insights into the lung of extinct coelacanths and the inner ear of the extant *L. chalumnae*. Our results suggest that, in extinct coelacanths, sound pressure was received by the ossified lung, transmitted via adjacent soft tissues, and conveyed to the inner ear through an extensive, unpaired perilymphatic system. Using synchrotron phase-contrast microCT, we show that two Triassic coelacanths, *Graulia branchiodonta* and *Loreleia eucingulata* gen. et sp. nov., possessed a multi-chambered ossified lung with anterodorsal bony projections attaching it to the notochord. Such ossifications may have facilitated the transmission of sound pressure from the lung via the notochord or other soft tissues. Based on synchrotron phase-contrast microCT and archival histological data of *L. chalumnae*, we reconstructed the complex three-dimensional architecture of the perilymphatic system, including a canal communicans, a canal superior, and a cochlear aqueduct. This perilymphatic system was first described in *L. chalumnae* by Millot and Anthony^12^. In fossil coelacanths, this system may have conveyed pressure-induced vibrations from the lung to the auditory sensory epithelia of the inner ear. These sensory epithelia, namely the basilar papilla^13^, a sarcopterygian autapomorphy^14^, and the amphibian papilla, present in most amphibians, would have transduced these vibrations via the endolymphatic space. Finally, comparative analysis with the Devonian coelacanth *Diplocercides kayseri* reveals a conserved perilymphatic organization, suggesting that this system may represent an ancestral auditory pathway in coelacanths.

## Results

### Lung anatomy of two fossil coelacanths *Graulia branchiodonta* and *Loreleia eucingulata* gen. et sp. nov

**Fig. 1:**
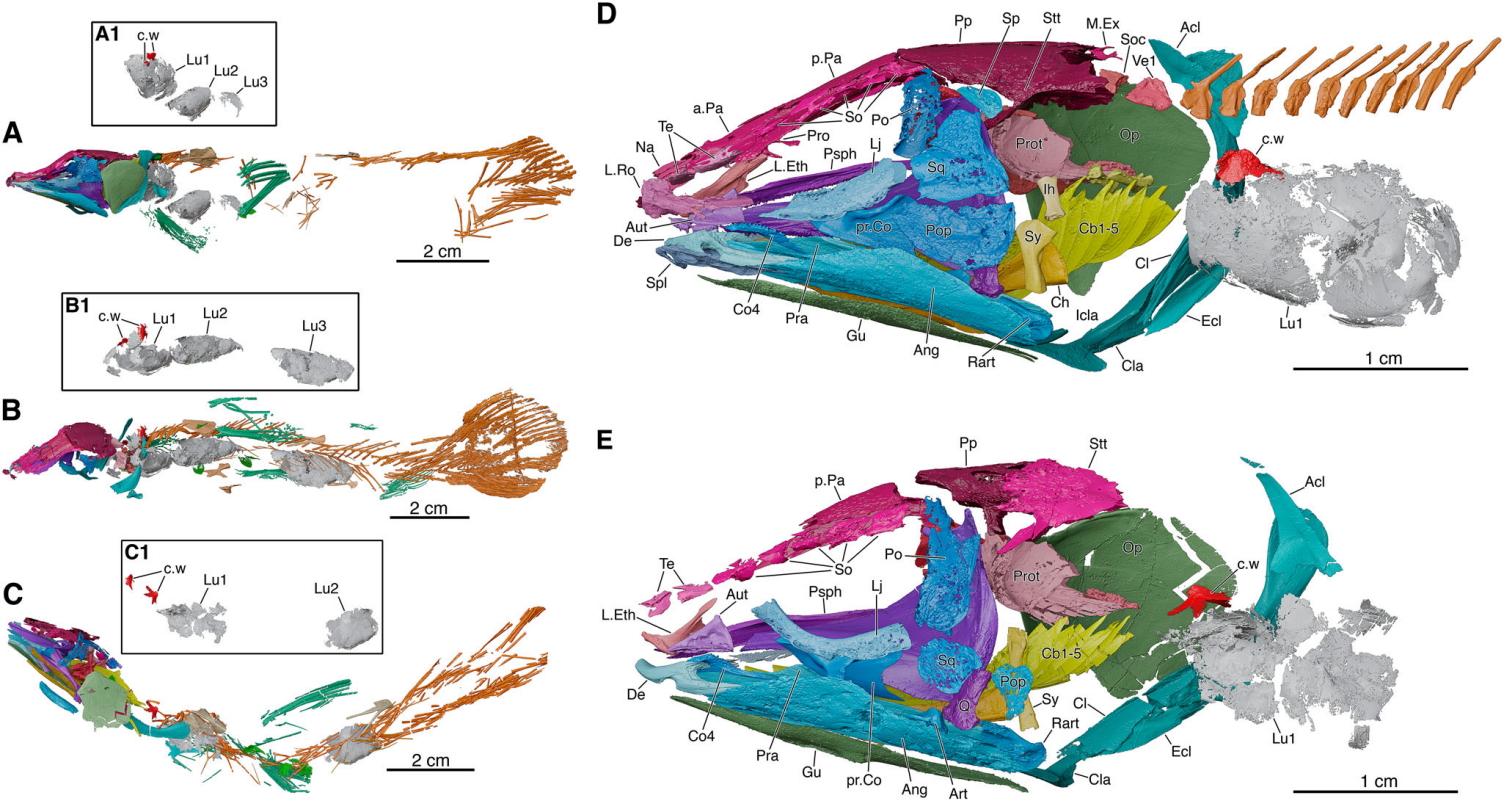
Skeletal anatomy of *Graulia branchiodonta* and *Loreleia eucingulata* gen. et sp. nov., based on synchrotron phase-contrast microCT. 360° rotation videos are available as Supplementary Movies 1 and 2. The nearly complete, three-dimensionally preserved skeletons of both taxa enabled a very detailed reconstruction. Each possesses a large, multichambered ossified lung bearing a pair of anterior dorsal crests (chamber wings). **A** *G. branchiodonta* holotype MHNG-GEPI-V5787. **B** *G. branchiodonta* referred specimen MHNG-GEPI-V5788. **C** *L. eucingulata* holotype MHNG-GEPI-V5789. (A1, B1, C1) Isolated ossified lungs corresponding to specimens in A, B, and C. **D** Reconstructed skull and anterior lung chamber of *G. branchiodonta* holotype. **E** Reconstructed skull and anterior lung chamber of *L. eucingulata* holotype. Abbreviations: Acl, anocleithrum; Ang, angular; a.Pa, anterior parietal; Art, articular; Aut, autopalatine; Cb1–5, ceratobranchials 1 to 5; Ch, ceratohyal; Cl, cleithrum; Cla, clavicle; Co4, fourth coronoid; c.w, chamber wing; De, dentary; Ecl, extracleithrum; Gu, gular plate; Icla, interclavicle; Ih, interhyal; L.Eth, lateral ethmoid; Lj, lachrymojugal; L.Ro, lateral rostral; Lu1–3, first to third lung chambers; M.Ex, median extrascapular; Na, nasal; Op, opercle; Po, postorbital; Pop, preopercle; Pp, postparietal; p.Pa, posterior parietal; Pra, prearticular; pr.Co, principal coronoid; Pro, preorbital; Prot, prootic; Psph, parasphenoid; Rart, retroarticular; So, supraocular; Soc, supraoccipital; Sp, spiracular; Spl, splenial; Sq, squamosal; Stt, supratemporal; Sy, symplectic; Te, tectal; Ve1, first vertebra. A high-resolution version of this figure can be downloaded from Figshare. (Figshare 10.6084/m9.figshare.31064614).

**Fig. 2:**
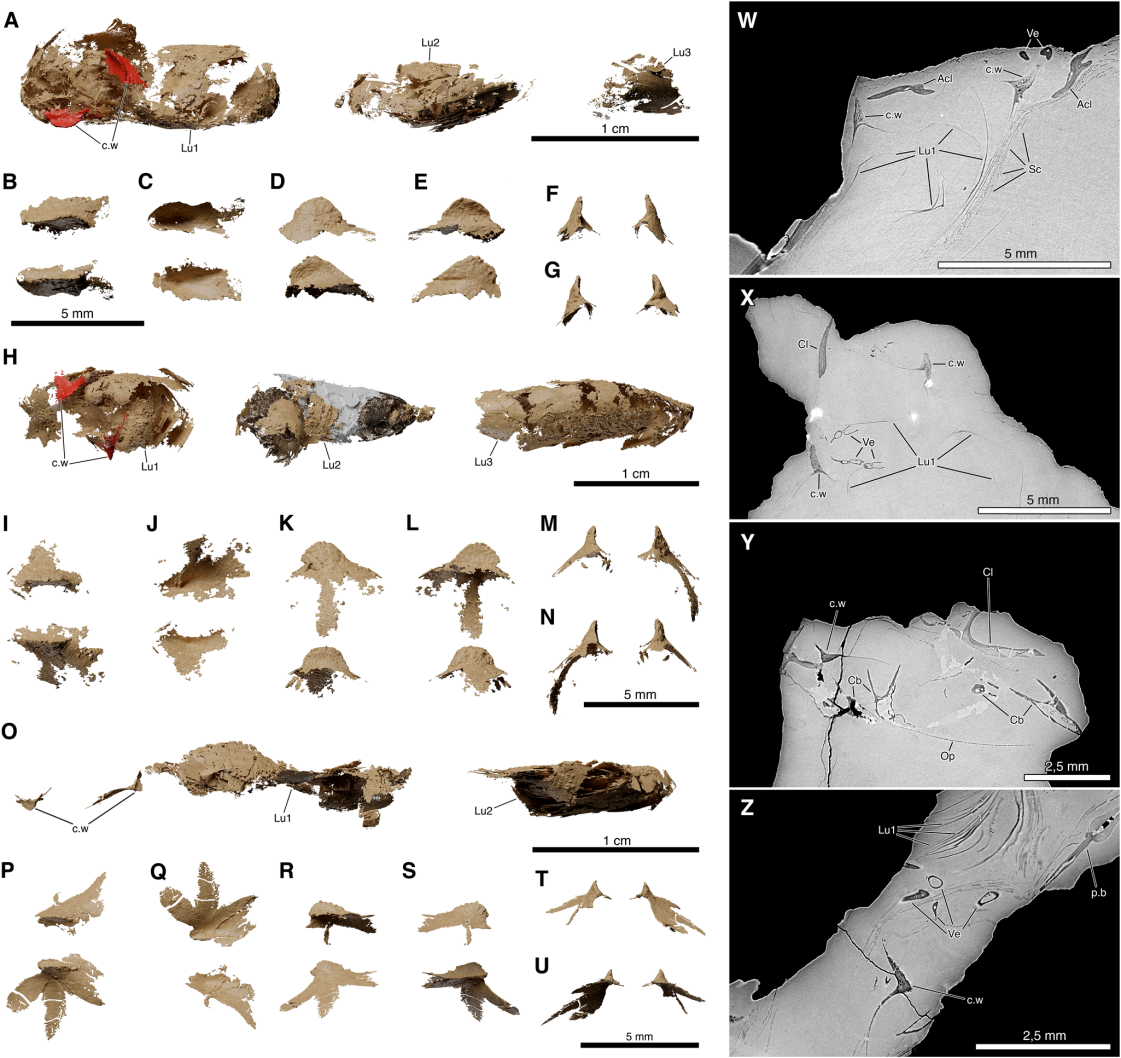
Ossified lung anatomy in *Graulia branchiodonta* and *Loreleia eucingulata* gen. et sp. nov., based on synchrotron phase-contrast microCT. Reconstructions show multi-chambered ossified lungs and paired dorsal crests (chamber wings) on the anterior chamber. Same specimens as in Fig. 1. **A** Dorsal view of the lung of *G. branchiodonta* holotype, showing chamber wings in situ. **B**–**G** Dorsal, ventral, left lateral, right lateral, anterior, and posterior views of the chamber wings from specimen in (**A**). **H** Dorsal view of the lung of the referred *G. branchiodonta* specimen with chamber wings in situ; high-density inclusions (metallic) and a lung cavity endocast (white) are visible in the second chamber. **I**–**N** Same as in (**B**–**G**) for the specimen in (**H**). **O** Dorsal view of the lung of the *L. eucingulata* holotype, showing chamber wings in situ. **P**–**U** Same as in (**B**–**G**) for the specimen in (**O**). **W**, **X** Tomographic sections through *G. branchiodonta* holotype and referred specimen. **Y**, **Z** Tomographic sections through the *L. eucingulata* holotype. Abbreviations: Acl, anocleithrum; Cb, ceratobranchial; Cl, cleithrum; c.w, chamber wing; Lu1–3, first to third lung chambers; Op, opercle; p.b, pelvic bone; Sc, scales; Ve, vertebrae. A high-resolution version of this figure can be downloaded from Figshare. (Figshare 10.6084/m9.figshare.31064614).

*Graulia branchiodonta*^15^ and *Loreleia eucingulata* gen. et sp. nov (formal taxonomic diagnosis in the Supplementary Note) are two latimerioid coelacanths from the Ladinian (Middle Triassic, ~240 Ma) of eastern France^15^ (Figs. 1 and 2, Supplementary Figs. 1 and 2). *G. branchiodonta* comprises two specimens, including the holotype, whereas *L. eucingulata* is represented by a single specimen, the holotype (see Methods for collection numbers). All of these specimens were studied here.

Each species is currently the only known representative of its genus. Both genera had a series of longitudinally arranged chambers enclosed by bony plates and probably filled with gas^1,6^, three in *Graulia* and two in *Loreleia*, located within the abdominal cavity. In *Graulia* and *Loreleia*, the chambers are covered by large yet very thin ossified plates arranged in a tile-like pattern. Due to incomplete preservation with some bony plates damaged or missing, it remains unclear whether these chambers were interconnected or connected to the digestive tract. The hypothesis of a connection to the digestive tract is supported by the known link between the oesophagus and the vestigial lung in the extant *L. chalumnae*^8^, considered homologous to the large ossified lung of fossil coelacanths, as well as by the presence of an anterior aperture in the lung of the fossil coelacanths *Macropoma*^3^ and *Undina*^16^ where the ossified plates are better preserved. In *Graulia* and *Loreleia*, for the first time observed in coelacanths, the anterior end of the most anterior chamber bears a dorsally positioned pair of specialized ossifications (Figs. 1, 2), here referred to as chamber wings. The absence of such features in the lung of other fossil coelacanths may be due to incomplete preservation or to the limitations of classical preparation techniques. Each wing consists of heterogeneous bone tissue with a denser outer layer and extends dorsally from the plate, forming a slightly longitudinally curved vertical ridge.

In our reconstruction, the chamber wings were ventrally attached to the external tunic of the notochord. No fossil evidence indicates the presence of ossifications forming a bony chain between the chamber wings and the neurocranium. While we cannot rule out that the chamber wings functioned merely to suspend the lung from the notochord, we favor the hypothesis of an auditory function. This interpretation is based on their location at the anterior end of the ossified chamber, near the perilymphatic system associated with the inner ear in *L. chalumnae* and likely in extinct coelacanths as well (Fig. 1D, E) (see below). To test this hypothesis and clarify the organization of the perilymphatic system in coelacanths, we examined the inner ear and associated auditory structures of the extant species *L. chalumnae*.

### Inner ear and accessory auditory structures in the extant coelacanth *Latimeria chalumnae*

**Fig. 3:**
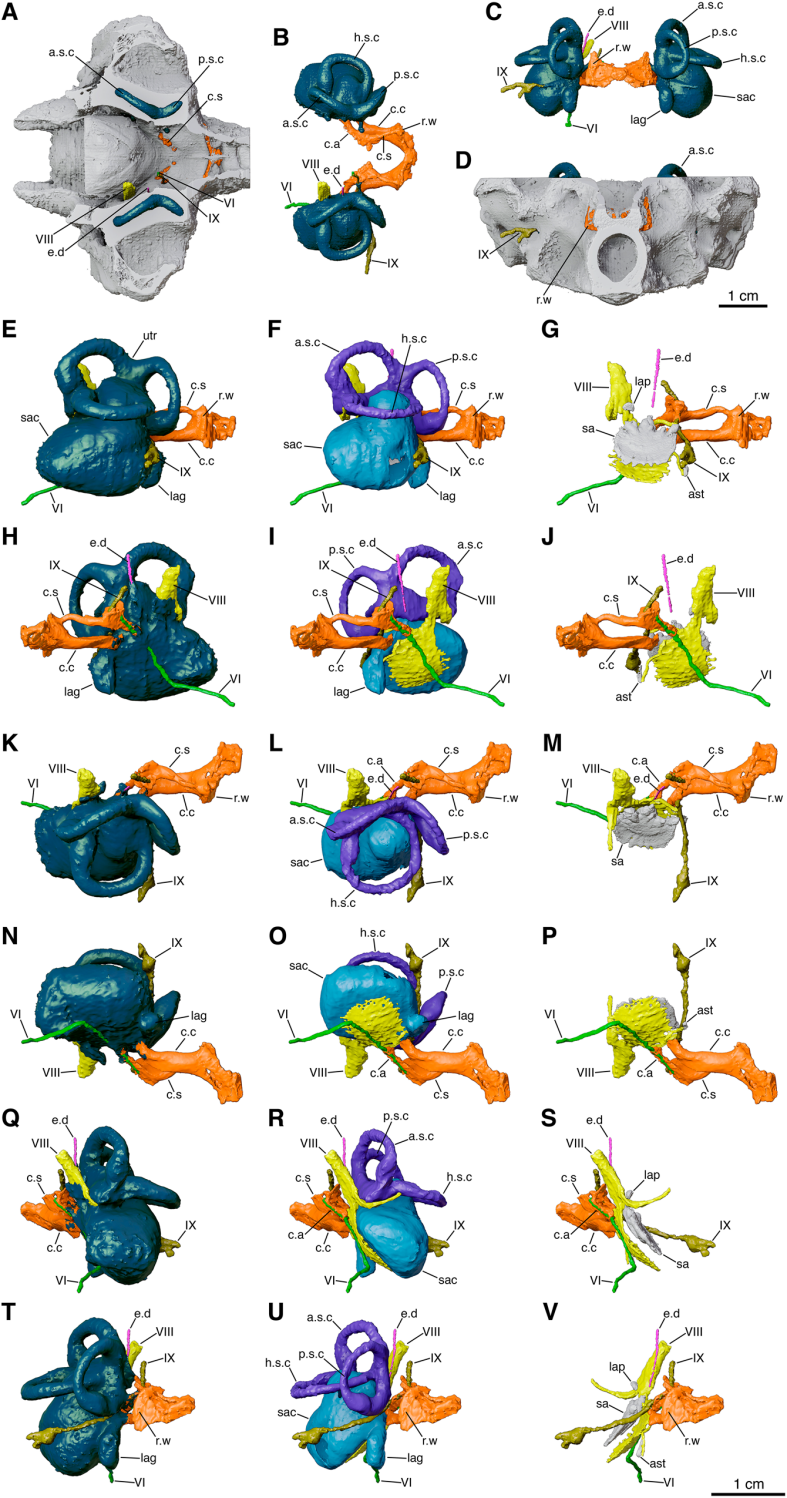
Inner ear anatomy of *Latimeria chalumnae* pups, revealed by synchrotron phase-contrast microCT. 360° rotation videos are available as Supplementary Movies 3 and 4. The reconstructions show the perilymphatic system, endolymphatic duct, and selected cranial nerves. The complex, unpaired perilymphatic system includes a canal communicans, a cochlear aqueduct and a canal superior. **A**–**D** Pup2, 356 mm TL, ZSMN-28409, CCC162.21, dorsal and posterior views of the otoccipital neurocranium, which is mostly cartilaginous, with visible openings of the inner ear cavities and associated canals. The skull roof has been removed; the brain is not shown. **B**, **C** Same views with the neurocranium removed. **E**–**V** Pup1a, 305 mm TL, MNHN-AC-2012-22, CCC29.5, isolated left inner ear shown in lateral (**E**–**G**), medial (**H**–**J**), dorsal (**K**–**M**), ventral (**N**–**P**), anterior (**Q**–**S**), and posterior (**T**–**V**) views. Progressive dissection reveals saccular and utricular membranes and otoliths. Abbreviations: a.s.c, anterior semicircular canal; ast, asteriscus; c.a, cochlear aqueduct; c.c, canal communicans; c.s, canal superior; e.d, endolymphatic duct; h.s.c, horizontal semicircular canal; IX, glossopharyngeal nerve; lag, lagena; lap, lapillus; p.s.c, posterior semicircular canal; r.w, round window; sa, sagitta; sac, saccule; VI, abducens nerve; VIII, vestibulocochlear nerve. A high-resolution version of this figure can be downloaded from Figshare. (Figshare 10.6084/m9.figshare.31064614).

We reconstructed the membranous labyrinth in 3D from synchrotron phase-contrast microCT, including otoliths and associated nerves, in a *L. chalumnae* pup specimen (Fig. 3E-V) (Pup1a, CCC29.5^17^). A large sagitta is present in the saccule^12^ and distinct small lapillus and asteriscus otoliths have been identified in the utricle and lagena, respectively. The macula of the sagitta is innervated by a ramified branch of the vestibulocochlear nerve (VIII), while the two smaller otoliths are each innervated by smaller branches of the same nerve^18^. The otolith organs detect sound waves, linear acceleration and static position in three-dimensional space^19^.

**Fig. 4:**
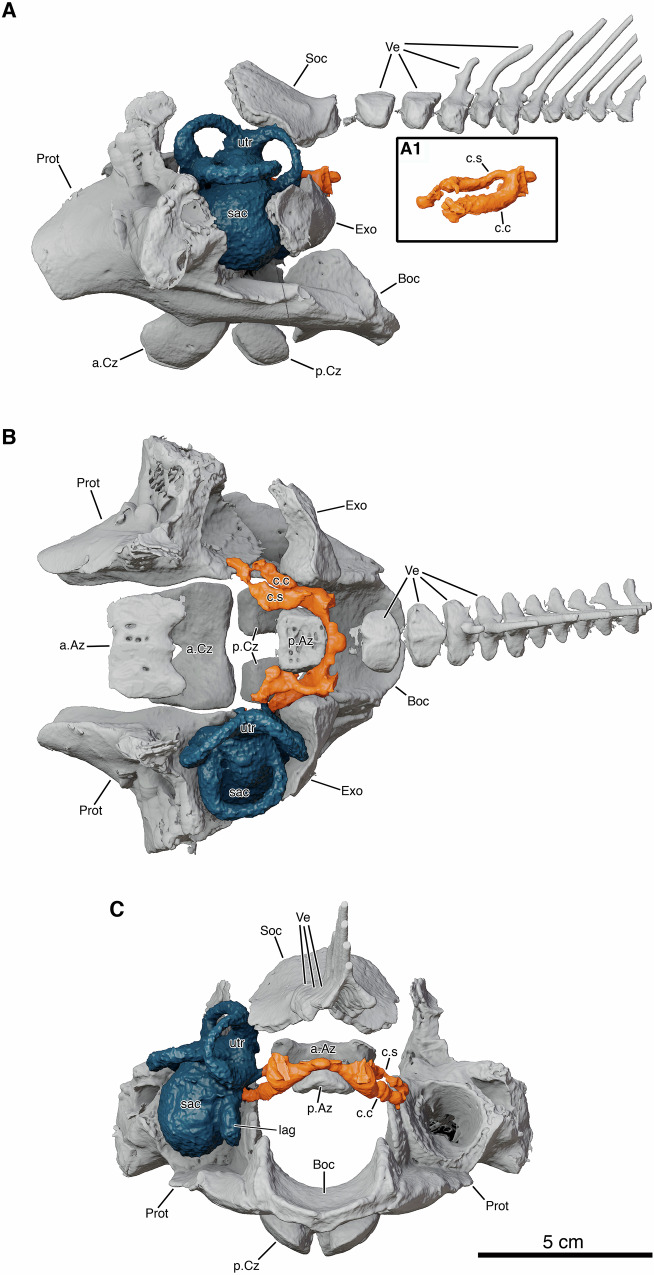
Otoccipital region of the neurocranium in *Latimeria chalumnae*, adult male, 1,300 mm TL, ZMUC-P1112, CCC23, based on standard microCT. Only ossified elements are shown, along with the cavities of the inner ear and perilymphatic system. The course and overall organization of the perilymphatic system in the adult matches that of pups, with the main difference being the increased ossification of neurocranial structures. The perilymphatic system canals contact the inner ear anteriorly, run between the exoccipitals and posterior anazygal, and merge posteriorly. The skull roof is omitted. Cavities of the perilymphatic system are slightly damaged. **A** Left lateral view. (A1) Isolated perilymphatic system, lateral view. **B** Dorsal view with supraoccipital removed. **C** Posterior view with exoccipitals removed. Abbreviations: a.Az, anterior anazygal; a.Cz, anterior catazygal; Boc, basioccipital; c.c, canal communicans; c.s, canal superior; Exo, exoccipital; lag, lagena; p.Az, posterior anazygal; p.Cz, posterior catazygal; Prot, prootic; sac, saccule; Soc, supraoccipital; utr, utricle; Ve, vertebrae. A high-resolution version of this figure can be downloaded from Figshare. (Figshare 10.6084/m9.figshare.31064614).

**Fig. 5:**
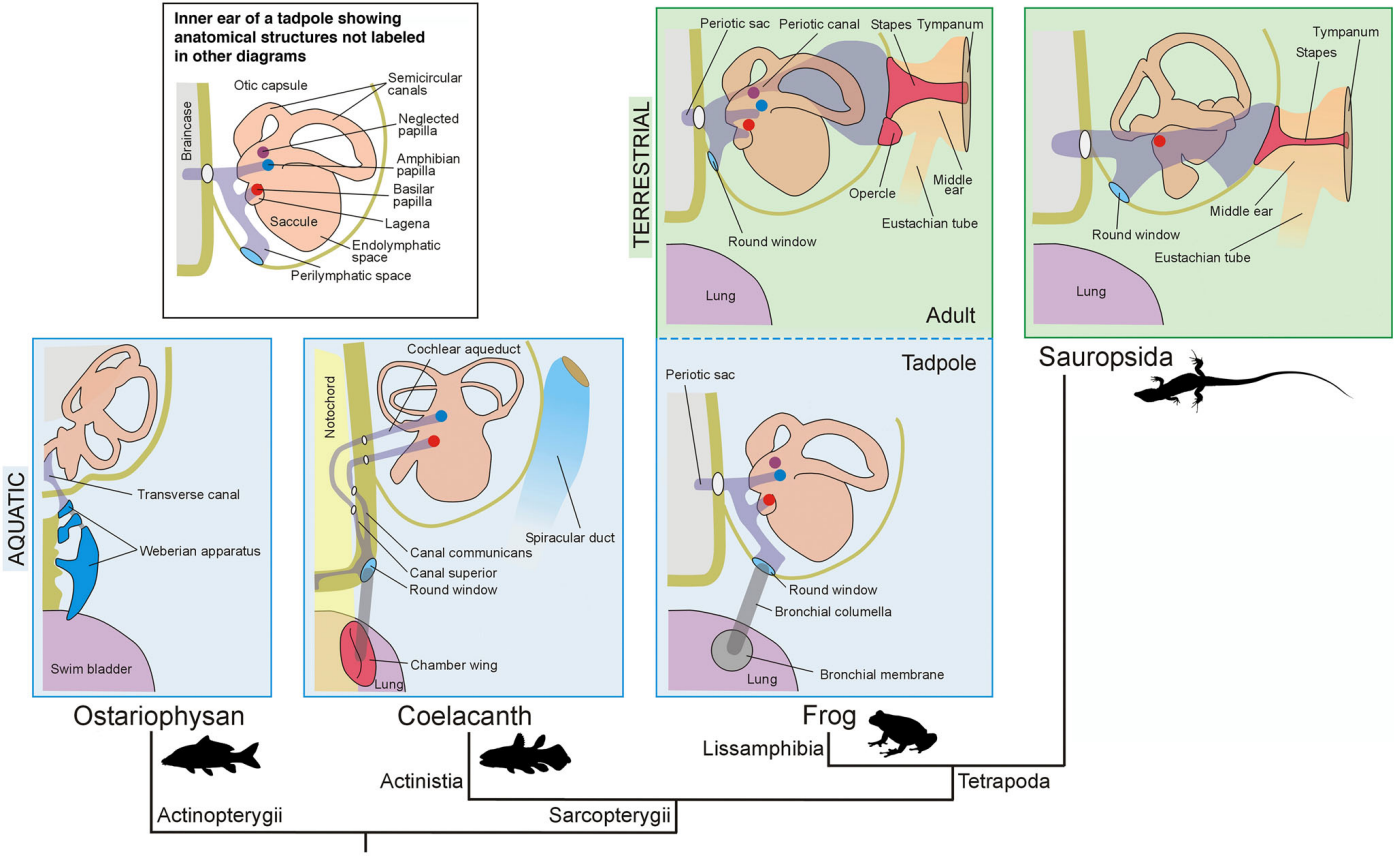
Relationships between gas-filled chambers and inner ear structures across osteichthyans. Comparative schematic of the connection between gas-filled chambers (lung or swim bladder) and the inner ear in selected osteichthyan lineages, shown in dorsal view of the right side and mapped onto a phylogeny. In aquatic taxa, sound pressure is transmitted from the gas-filled chamber via bones, ligaments, or soft tissues to perilymphatic canals relayed to the inner ear. In terrestrial vertebrates, this connection is absent; instead, a tympanic membrane and stapes transmit airborne sound to the inner ear via the perilymphatic space. The schematic for the coelacanth is putative and is intended to represent extinct forms, based on evidence from both extant and fossil taxa (see also Fig. 6). A high-resolution version of this figure can be downloaded from Figshare. (Figshare 10.6084/m9.figshare.31064614).

From standard and synchrotron phase-contrast microCT, we reconstructed in 3D the perilymphatic system in two *L. chalumnae* pups (Pup1a and Pup2) (Fig. 3, Supplementary Fig. 3) and in one adult (Fig. 4), showing that its overall arrangement remains consistent. Archival histological sections of a third pup (Pup1b) (Supplementary Figs. 4-6) indicate that our observations partially differ from previous descriptions^12,20–22^. Fritzsch^13^ identified a basilar papilla, homologous to that of amphibians and amniotes, at the boundary between the saccule and the lagena. This papilla rests on a basilar membrane that separates the endolymphatic and perilymphatic spaces. Our reconstruction slightly diverges from Fritzsch et al. ^14^ where the perilymphatic space near the basilar papilla was described as leading directly to the cerebral cavity. Instead, our 3D reconstruction shows the perilymphatic space extending posteriorly through the prootic cartilage forming the canal communicans, a structure first described by Millot and Anthony^12^ (see below). Another canal described by Fritzsch et al. ^13^ which crosses the cartilage separating the inner ear from the cerebral cavity, is present but contacts the saccule anterodorsally relative to the basilar papilla. Histological sections and CT data reveal that this short canal, homologous to the cochlear aqueduct of tetrapods^14^, accommodates both fluid and the abducens nerve (VI) (Fig. 3, Supplementary Figs. 3-6). At the connection point between the cochlear aqueduct and membranous labyrinth, slightly ventral to the utriculo-saccular foramen, a second papilla is present. This papilla was previously considered the “neglected papilla” by Fritzsch^13^, but given its direct association with the perilymphatic space, it can also be regarded as homologous to the amphibian papilla found in amphibians. It is possible that the neglected papilla shifted slightly during evolution, as observed in gymnophionans^23^, to facilitate a more direct connection to the perilymphatic space. Here, we refer to this structure in *L. chalumnae* as the amphibian papilla, recognizing that both the neglected and amphibian papillae share the same evolutionary origin^23^. The presence of two auditory papillae, the basilar and amphibian papillae, both associated with the perilymphatic space, mirrors the arrangement observed in amphibians (Fig. 5).

In the brain cavity, just posterior to the opening of the cochlear aqueduct, a second canal emerges that encloses the glossopharyngeal nerve (IX) and lies in contact with the cochlear aqueduct. This nerve enters the inner ear cavity, then courses posteriorly, bypassing the membranous labyrinth, before piercing the prootic cartilage and exiting the skull laterally. On the floor of the cerebral cavity, just posterior to the foramen of nerve IX, a groove extends posteriorly, bordered laterally by the prootic cartilage and medially by a thickening of the external tunic of the notochord. No soft membrane appears to separate the lumen of this canal from the brain cavity, making its interpretation uncertain. Further posteriorly, this groove transitions into a fully enclosed canal surrounded by cartilage, refer to here as the canal superior (Figs. 3 and 5, Supplementary Fig. 3). This canal runs almost parallel to the canal communicans, located ventrally. The canal communicans, after leaving the basilar papilla, extends posteriorly, bordered medially by the thickened external tunic of the notochord and laterally by the prootic cartilage, before continuing entirely within the cartilage. The two canals merge within the prootic, and the resulting canal bends at the posterolateral corner of the skull, meeting its counterpart at the midline. At this turn, the canal communicans opens laterally through a fenestra in the cartilaginous braincase, where a round window was likely present. In the pups, the posterior portions of the canal communicans, including the commissure, are filled with connective tissue (Supplementary Fig. 6). This tissue connects to the outer connective tissue of the braincase through the paired lateral openings described above. As a result, the perilymphatic space in *L. chalumnae* may have lost its auditory function, which is expected given the vestigial state of the lung.

## Discussion

**Fig. 6:**
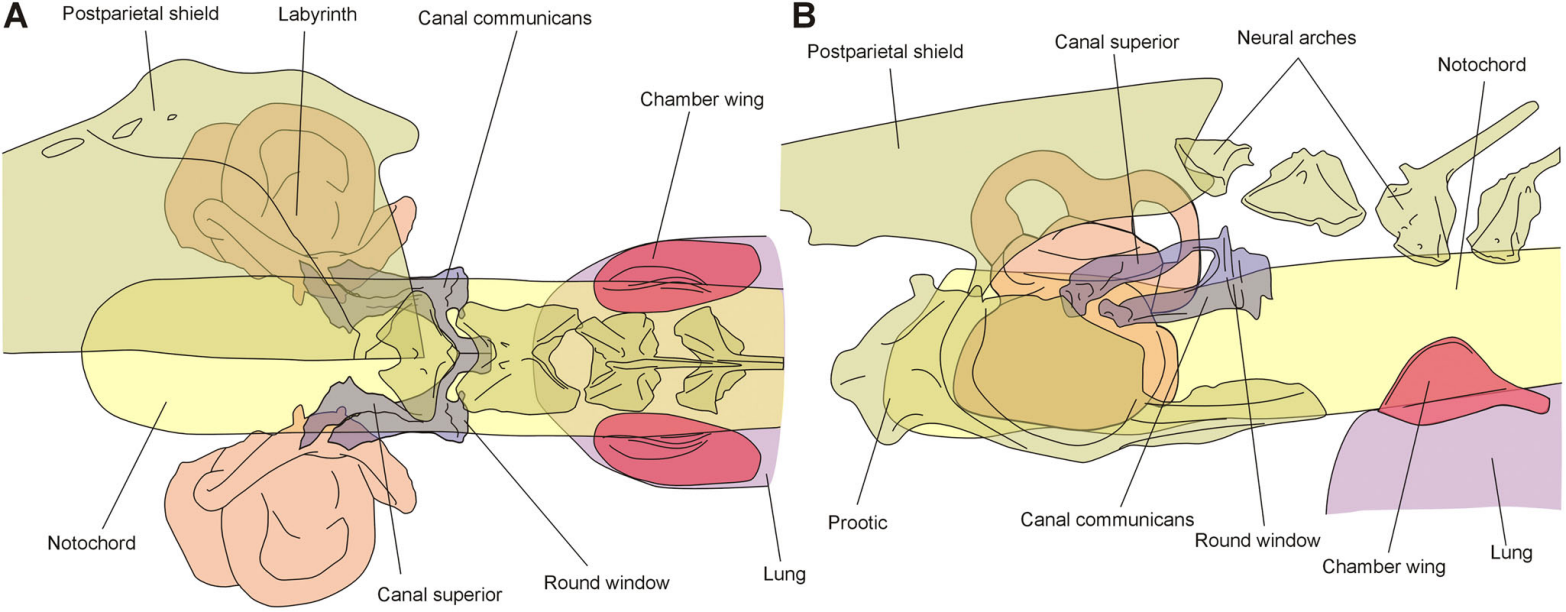
Reconstruction of putative auditory structures in extinct coelacanths. **A** dorsal, **B** left lateral views. Skeletal components are based on *Graulia* (see Figs. 1–2) while inner ear and perilymphatic anatomy follows *L. chalumnae* (see Figs. 3–4). The ossified lung bears dorsal ridges (chamber wings) hypothesized to transmit sound vibrations to the inner ear via the notochord or other soft tissue, through a perilymphatic system comprising the canal communicans and canal superior. Both the lung and the perilymphatic system are vestigial in *L. chalumnae*, so their auditory function in extinct coelacanths can only be inferred by integrating data from extant and fossil taxa. A high-resolution version of this figure can be downloaded from Figshare. (Figshare 10.6084/m9.figshare.31064614).

The original function of the perilymphatic system of *L. chalumnae* has been debated since its discovery^12,20,22^. Millot and Anthony^12^ noted its resemblance to the perilymphatic spaces associated with the inner ear of otophysan teleosts with a Weberian apparatus, that is to say a perilymphatic sinus impair that transmits sound vibrations from the scaphium, the most anterior ossicle of the Weberian apparatus. The question remained unresolved, as no anatomical structures comparable to the Weberian apparatus, nor any gas-filled chambers, had been observed in *L. chalumnae*. Here, we support the hypothesis that the perilymphatic accessory structures (canal communicans, canal superior and cochlear aqueduct) observed in *L. chalumnae* served an auditory function in extinct coelacanths that has been lost along the *L. chalumnae* lineage, a possibility already noted by Millot and Anthony^12^. Our interpretation is based on the association of the perilymphatic accessory structures with two sensory epithelia, the basilar and amphibian papillae, which are involved in sound pressure detection in amphibians, rather than with structures related to balance^14,22^. In the fossil coelacanths *Graulia* and *Loreleia*, the ossified portion of the prootic encapsulates only the anteroventral part of the otic capsule^15^ (Fig. 6). Consequently, the membranous labyrinth and perilymphatic system, enclosed within the cartilaginous mass of the occipital region, remain unknown in these genera. In contrast, the otico-occipital region of the heavily ossified Late Devonian *Diplocercides kayseri* (*Nesides schmidti*, synonymized by Forey^24^) is relatively well understood, thanks to a wax model reconstruction made and described first by Stensiö^25^ (Supplementary Fig. 7). Although Bjerring^21,26^ provided a slightly different interpretation, the courses of the cranial nerves in relation to the otic capsule appear consistent with *L. chalumnae*. A perilymphatic system structurally similar to that of *L. chalumnae* is recognized in *D. kayseri*, featuring a cochlear aqueduct (referred to as the “canal for the abducens nerve“^26^, this canal accommodates both the abducens nerve and the cochlear aqueduct in *L. chalumnae*) and a large canal communicans (termed the “endolymphatic occipital commissure“^26^). This canal communicans runs transversely across the posterior wall of the occipital region, between the foramen magnum dorsally and the notochordal canal ventrally. Bjerring^26^ also described in *D. kayseri* a connection between the brain cavity and the canal communicans (“canal for tubular branch of occipital commissure”), which we interpret as homologous to the canal superior described in *L. chalumnae*. Finally, the “foramen between canal for notochord and space for endolymphatic occipital commissure” described by Bjerring^26^ may correspond to the contact between the canal communicans and the surface of the notochord observed here in *L. chalumnae*. Given the presence of a perilymphatic system with a canal communicans in both *L. chalumnae* and *D. kayseri*, a basal coelacanth^2^, we infer through phylogenetic bracketing that this specialized perilymphatic system is an apomorphy of coelacanths. Consequently, it is likely that a similar system was also present in *Graulia* and *Loreleia*.

The presence of potential sound-conveying ossifications on the lung of fossil coelacanths, along with a posterior extension of the perilymphatic system oriented toward the exterior of the skull, is reminiscent of the Weberian apparatus and perilymphatic system in otophysans (Fig. 5). This similarity reflects evolutionary convergence rather than homology. Unlike otophysans, coelacanths lack a chain of ossicles linking the gas-filled chamber to the inner ear. Additionally, in otophysans, sound pressure is detected via the saccular and lagenar otoliths, whereas in coelacanths, similar to tetrapods, detection likely occurred through the basilar and amphibian papillae. The organization of the perilymphatic space in *L. chalumnae* closely resembles that of anuran tadpoles, as both have a dual connection to two sensory epithelia: the basilar and amphibian papillae^27^ (Fig. 5). More specifically, the inner ear of *L. chalumnae* lacks a periotic canal leading to the periotic cistern, which, in adult anurans, transmits sound vibrations from the stapes. However, in tadpoles, the periotic cistern is absent, and the tympanic membrane is nonfunctional^14,28^, resulting in a condition similar to *L. chalumnae*^22^. In anuran tadpoles, a pair of bronchial membranes on the lung surface connect to the round windows by a tendon-like structure, the bronchial columella^28^. Although the connection between this columella and the inner ear in tadpoles remains poorly studied and its function debated^29^, it is possible that in *Graulia* and *Loreleia*, a fibroblastic and collagenous element comparable to the tadpole’s bronchial columella transmitted sound vibrations from the chamber wings to the perilymphatic system^22^. This transmission would occur where the canal communicans opens laterally to the skull. However, the presence of specialized structures linking the lung to the neurocranium is not necessarily required for sound pressure detection. For instance, extant lungfish can detect sound pressure via their lungs in water, through the substrate, and in air, though without a direct lung–inner ear connection^9^. In fossil coelacanths, the chamber wings may have primarily served to anchor the lung to the notochord near the neurocranium, thereby allowing the transmission of pressure-induced vibrations to the perilymphatic system through adjacent tissues. Vibrations may have travelled along the solid yet elastic notochord, which anteriorly contacts both the canal superior and the canal communicans near their junction with the endolymphatic space (Fig. 6). Overall, the organization of the inner ear and perilymphatic space in *L. chalumnae and D. kayseri*, combined with lung anatomy in *Graulia* and *Loreleia*, suggests a putative auditory role for this complex apparatus in extinct coelacanths, in addition to its likely respiratory function. Within this framework, the reduction of the lung and the infilling of the commissural region of the canal communicans with connective tissue in the lineage leading to *L. chalumnae* may reflect the loss of both respiratory and auditory roles of the lung. These new anatomical findings support a scenario similar to that proposed by Millot and Anthony^12^.

Paleontological evidence across vertebrate evolution provides few clues about the origin and function of the air-filled chamber in osteichthyans. However, physiological and genetic studies suggest that lungs and swim bladders are homologous, with lungs possibly serving an initial respiratory function before the evolution of swim bladders primarily involved in buoyancy regulation^30,31^. Coelacanths may play a role in understanding the evolution of this organ. They represent the earliest-diverging living lineage of sarcopterygians and retain some ancestral traits of the clade considered to be partly due to their slow morphological evolution^32^. A respiratory role for the lung is consistent with its atrophied condition in *L. chalumnae*, which has been interpreted as an adaptation to life in moderately deep marine environments^5^. Additional support comes from the lung of a Cretaceous coelacanth, which shows a vascular system organized similarly to that of the gas-filled chamber of extant air-breathing fishes^7^. A potential auditory role for the coelacanth lung is suggested by the presence of a perilymphatic system in a Devonian coelacanth, comparable to that of *L. chalumnae*^21,26^, and by the presence of distinct gas-filled chambers in some extinct coelacanths. This arrangement recalls the condition observed in otophysan fishes, which possess an anterior *camera aerea weberiana* associated with sound transmission^19^. Such clues raise the possibility that hearing via sound transmission from air within the lung constituted an early sensory mechanism in coelacanths, and potentially in sarcopterygians more broadly. The development of auditory papillae in association with a complex perilymphatic system may predate the evolution of the middle ear and tympanum in tetrapods. Consequently, early sarcopterygians may have possessed inner ear organs capable of detecting airborne sound, without tympanic specialization, prior to the transition to land.

## Methods

### Specimens examined

The specimens studied are housed in the American Museum of Natural History (AMNH), the Muséum National d’Histoire Naturelle (MNHN), the Natural History Museum of Geneva (MHNG), the Zoological Museum of the University of Copenhagen (ZMUC), and the Zoologische Staatssammlung München (ZSMN). The holotype of the fossil coelacanth *Loreleia eucingulata* (MHNG-GEPI-V5789) (Fig. 1C,E, Supplementary Figs. 1D-F,K-M and 2A) comprises a nearly complete body, including the complete skull, axial skeleton, and relatively complete paired and median fins, with an estimated total length (TL) of 145 mm. The specimen is likely a juvenile, the adult stage is not known. The holotype of the fossil coelacanth *Graulia branchiodonta* (MHNG-GEPI-V5787) and the referred specimen (MHNG-GEPI-V5788) both exhibit a nearly complete body, including the complete skull, axial skeleton, and relatively complete paired and median fins, with an estimated TL of 160 mm. These specimens are also likely juveniles, no adult stage is currently known. For further information regarding the fossil *Graulia branchiodonta* specimens, see ref.^15^. Specimens of the extant coelacanth *Latimeria chalumnae* include Pup1a (MNHN-AC-2012-22, *CCC29.5*), a 305 mm TL pup with a yolk sac; Pup1b (AMNH-32949h, *CCC29.1*), a 303 mm TL pup with a yolk sac; Pup2 (ZSMN-28409, *CCC162.21*), a 356 mm TL late pup without a yolk sac; and an adult male (ZMUC-P1112, *CCC23*) measuring 1300 mm TL. Pup1a, Pup2 and the adult male are preserved in ethanol. Only serial sections of Pup1b are available from the AMNH. Pup1a and Pup1b come from the same litter. For further information regarding *L. chalumnae* specimens see ref.^17^.

### Data acquisition

The fossil coelacanths *Loreleia eucingulata* (MHNG-GEPI-V5789) and *Graulia branchiodonta* (MHNG-GEPI-V5787, V5788), as well as the extant species *L. chalumnae* (Pup1a, Pup2) were imaged with Propagation Phase Contrast Synchrotron Radiation micro-Computed Tomography (PPC-SRµCT) at the European Synchrotron and Radiation Facility (ESRF), Grenoble, France. The fossil specimens were imaged on beamline BM05 (10.15151/ESRF-ES-788657609) using a filtered white beam and a 2000 nm LuAG scintillator, with a 3.5 m sample–detector distance and an isotropic voxel size of 14.9 µm. MHNG GEPI V5788 was scanned at 117.5 keV with molybdenum (2.16 mm) and aluminium (4.47 mm) filters, using an exposure time of 120 ms (4 × 30 ms) over 6000 projections. MHNG GEPI V5787 and V5789 were scanned at 110 keV with a copper (6.41 mm) filter, with an exposure time of 27 ms (3 × 9 ms) over 6000 projections. Datasets were acquired with a PCO edge 4.2 sCMOS detector in accumulation mode and a half-acquisition setup with a 900-pixel offset. For a detailed account of the scan parameters of the fossil coelacanths, see ref.^15^. The tomographic scans of *L. chalumnae* were acquired in previous studies^33–35^ and are available from public repositories. PPC-SRµCT scans of the neurocranium of *L. chalumnae* Pup1a (MNHN-AC-2012-22, *CCC29.5*) and Pup2 (ZSMN-28409, *CCC162.21*) were downloaded from the ESRF Paleo Database (https://paleo.esrf.fr/datasets/1634387924) (10.15151/ESRF-DC-1634387693), while the CT scan of the adult male *L. chalumnae* was obtained from Morphosource (Media 000398327). The *L. chalumnae* specimens Pup1a and Pup2 were scanned at beamline ID19. The specimens were imaged with an isotropic voxel size of 30.45 µm, an average energy of 63.2 keV, and a propagation distance of 2800 mm. The filter setup included aluminium (2 mm), copper (0.25 mm), and tungsten (0.25 mm). Data acquisition was completed with a FReLoN 2K14 sensor over 4998 projections in half-acquisition mode, with a 700 µm LuAg: Ce scintillator installed. The exposure time was 0.1 s per projection. All tomographic volumes from the ESRF were reconstructed in PyHST2^36^ using Paganin single-distance phase retrieval^37^. The adult *L. chalumnae* specimen (ZMUC-P1112) was imaged using a Siemens Somatom Definition Dual Energy CT system^35^. Scanning was performed at 120 kVp and 428 mA, with an integration time of 1000 ms. The acquisition covered a field of view of 102.0 × 102.0 × 207.6 mm³, with an isotropic spatial resolution of 0.2 mm. A B45s convolution kernel was applied during reconstruction. For further details on data acquisition on *L. chalumnae* Pup1a and Pup2, see the original publication^33,34^, and for the adult male specimen, see ref.^35^. The scans of the archival histological sections of Pup1b were provided by the American Museum of Natural History, Ichthyology Department.

### Segmentation and rendering

MicroCT datasets were manually segmented in Dragonfly 4.1 (Comet). Each anatomical structure was segmented manually using local thresholding. Individual meshes were exported as PLY files and imported into Blender v3.1.2 (Blender Institute). In Blender, the skeletal elements of the fossil skulls were manually repositioned by attempting to reconnect each bone in its putative natural position, guided by the articulations observed in a 3D reference model of the skull of the extant species *L. chalumnae*^38^. Final 3D models were rendered in Blender using two different methods. For images with colorized anatomical structures, the Workbench render engine was used; each element was vertex-colorized, convexities and concavities were enhanced, and contours were highlighted with a thin black outline. For photorealistic bone-like textures, the Cycles render engine was employed, incorporating a complex shading pattern with several nodes, including a Noise Texture with a ColorRamp (ranging from brown to white). Ambient occlusion was applied in some renders to emphasize cavities. A consistent lighting setup was used for all renderings, consisting of a strong, sun-like light source from the upper left corner and a softer point light from below to diffuse shadows. Rendered images were exported as TIFF files and refined in Adobe Photoshop 2021 to adjust exposure and combine multiple images into final figure plates. Our figures are designed according to standard ichthyology conventions, as follows. Dorsal, ventral, left lateral, right medial views: anterior is to the left. Right lateral, left medial views: anterior is to the right. Anterior and posterior views: the top of the figure corresponds to the dorsal side.

### Phylogenetic analysis

Our data matrix comprises 50 taxa and 112 characters (Supplementary Data 1). It is based on the recently published coelacanth data matrix of ref.^39^. Character 66 (sub-opercular branch of the mandibular sensory canal: (0 absent / 1 present)) was removed from the analysis, following the recommendations of ref.^15^. Parsimony analyses were conducted in PAUP* 4.0^40^ using heuristic searches with a random stepwise addition sequence (100 replicates), holding 10 trees at each step. Tree-bisection-reconnection (TBR) branch swapping was employed, swapping only on the best trees. Statistical support was assessed using bootstrap values.

### Nomenclatural acts

This published work and the nomenclatural acts it contains have been registered in ZooBank, the proposed online registration system for the International Code of Zoological Nomenclature (ICZN). The ZooBank LSIDs (Life Science Identifiers) can be resolved and the associated information viewed through any standard web browser by appending the LSID to the prefix “http://zoobank.org/”. The LSIDs for this publication are:

urn:lsid:zoobank.org:act:7130D7EF-B054-497F-AE4E-C9B6502D6EBA

urn:lsid:zoobank.org:act:739805E7-2596-4072-9F0C-4474E0BC4282

## Supplementary information

Supplementary Information

Description of Additional Supplementary File

Supplementary Data 1

Supplementary Movie 1

Supplementary Movie 2

Supplementary Movie 3

Supplementary Movie 4

Reporting-summary

## Data Availability

The synchrotron scan files of *Graulia branchiodonta* (MHNG-GEPI-V5787, holotype and MHNG-GEPI-V5787, referred specimen) are available from the ESRF Paleontology Database (https://paleo.esrf.fr/datasets/2015882168 ; https://paleo.esrf.fr/datasets/2015882170)^41,42^. The synchrotron scan files of *Loreleia eucingulata* (MHNG-GEPI-V5789, holotype) will be publicly available from the ESRF Paleontology Database (https://paleo.esrf.fr/) along with surface files of the individual bones. The fossil material housed in the Natural History Museum of Geneva is available for study upon request.
