# Supplementary material for: A dual respiratory and auditory function for the coelacanth lung
Source: Commun Biol. 2026 Feb 14;9:400. doi: 10.1038/s42003-026-09708-6 (PMC13003144; doi:10.1038/s42003-026-09708-6)
Supplement: Supplementary file 1 — Supplementary Information [file 42003_2026_9708_MOESM1_ESM.pdf]

A dual respiratory and auditory function for the coelacanth lung

Luigi Manuelli[1,2]*, Gaël Clément[3], Marc Herbin[4], Bernd Fritzsch[5], Per E. Ahlberg[6], Kathleen Dollman[7], Lionel Cavin[1,2]

[1]Department of Earth Sciences, Natural History Museum of Geneva; Geneva, Switzerland.

[2]Department of Genetics and Evolution, University of Geneva; Geneva, Switzerland.

[3]Centre de Recherche en Paléontologie-Paris (CR2P, MNHN-CNRS-Sorbonne Université), Muséum National d'Histoire Naturelle; Paris, France.

[4]Mécanismes Adaptatifs et Evolution (MECADEV, MNHN-CNRS-Sorbonne Université) Muséum National d'Histoire Naturelle; Paris, France.

[5]Department of Neuronal Sciences, University of Nebraska Medical Center; Omaha, USA.

[6]Department of Organismal Biology, Uppsala University; Uppsala, Sweden.

[7]European Synchrotron and Radiation Facility; Grenoble, France.

*Corresponding author. Email: Luigi.manuelli@unige.ch

This document includes:

Supplementary Note page 1
Supplementary Figs. 1-7 page 4

Supplementary Note

Systematic paleontology

Class OSTEICHTHYES Huxley, 1880
Subclass SARCOPTERYGII Romer, 1955
Infraclass ACTINISTIA Cope, 1891
Order COELACANTHIFORMES Huxley, 1861
Suborder LATIMERIOIDEI Schultze, 1993
Incertae sedis

Loreleia eucingulata gen. et sp. nov. Manuelli & Cavin

urn:lsid:zoobank.org:act:7130D7EF-B054-497F-AE4E-C9B6502D6EBA
urn:lsid:zoobank.org:act:739805E7-2596-4072-9F0C-4474E0BC4282

Etymology

The genus name *Loreleia* refers to the Loreley, a mythical figure from the folklore of Lorraine, the region of France where the specimens were found. The species name *eucingulata* derives from the Greek prefix "eu-" (εὖ), meaning "well" or "prominent," and the Latin "cingulata" (*cingulum*), meaning "girdle/belt." It refers to the elongated pectoral girdle of the species.

Holotype and only known specimen

Natural history museum of Geneva (MHNG), an articulated and nearly complete skeleton (MHNG-GEPI-V5789, Figs. 1 and 2, Supplementary Figs. 1, 2 and Movie 2).

Locality and horizon

Sarraltroff, Moselle, Grand Est, France. Middle Triassic Muschelkalk, Calcaire à Cératites Formation, *Ceratites praenodosus* biozone (a ceratite ammonoid) (Jean-Philippe et Francois-Xavier Blouet, personal communication 2018). The *praenodosus* zone from France corresponds to the Tonhorizont epsilon from Germany, which belongs to the Hohenhole Formation from the Upper Muschelkalk, middle Ladinian (Fassanian, 242–237 Mya), early Middle Triassic. A detailed description of the geological setting will be published in a future article.

Diagnosis of genus and species

Latimerioid coelacanth characterized by the following association of characters (characters used in the phylogenetic analysis are marked with an asterisk (*)): parasphenoid with a mid-length constriction (proportionally wider in *Graulia*); *parasphenoid with an anterior ascending lamina (absent in *Graulia*);*supraorbital sensory canals opening as many pores within bones (larger and less numerous in *Graulia*); *spiracular absent (present in *Graulia*); *squamosal reduced limited to the mid-level of cheek (large and extending dorsally in *Graulia*); *preopercle small and simple and located posteriorly to the squamosal (large, with an anterior blade-like portion and located below the squamosal in *Graulia* and *Dobrogeria*[1]; *subopercle absent (present in *Graulia* and *Dobrogeria*); small ectopterygoid (larger in *Graulia* and *Dobrogeria*); gular narrow (wide in *Graulia* and *Dobrogeria*); coronoid elongated, with a well-developed process forming a bridge medially with the angular (shorter, without process in *Graulia*); elongated and narrow sensory pores on the angular (broader in *Graulia*); urohyal long and narrow (short and wide in *Graulia*); ceratobranchial with small teeth (with large, fang-like teeth in *Graulia*); cleithrum very elongated, slightly curved (shorter and more angled in *Graulia*, incomplete but apparently only slightly curved in *Dobrogeria*).

Remark on the diagnosis

Our phylogenetic analysis (Supplementary Fig. 2B) places *Loreleia eucingulata* in a polytomy with *Graulia branchiodonta*[2], found in the same locality, and *Dobrogeria aegyssensis*[1], a poorly known species from the Lower Spathian of Northern Dobrogea, Romania. A detailed description of *L. eucingulata* will be provided elsewhere. The diagnosis presented here is comparative,

emphasizing characters that distinguish *L. eucingulata* from *Graulia* (Supplementary Fig. 1) and *Dobrogeria*, where data are available.

Remark on the phylogeny

In previous phylogenetic analyses, *Graulia branchiodonta* was identified as the most basal mawsoniid. However, our results place *Graulia*, along with *Loreleia* and *Dobrogeria*, as the earliest diverging latimeriids, preceding the split between Latimeriinae and Ticinepomiinae. This section of the coelacanth phylogeny, comprising the most basal mawsoniids and latimeriids, remains labile, with taxonomic placements varying between studies. As this study does not primarily focus on resolving the phylogeny of the group, we provisionally consider *Graulia*, *Loreleia*, and *Dobrogeria* as basal Latimeroidei *incertae sedis*, pending further analyses.

Supplementary References

1. Cavin, L. & Grădinăru, E. *Dobrogeria aegyssensis*, a new early Spathian (Early Triassic) coelacanth from North Dobrogea (Romania). *Acta Geol. Pol.* **64**, 139–165 (2014).
2. Manuelli, L., Mondéjar Fernández, J., Dollman, K., Jakata, K. & Cavin, L. The most detailed anatomical reconstruction of a Mesozoic coelacanth. *PLOS ONE* **19**, 0312026 (2024).

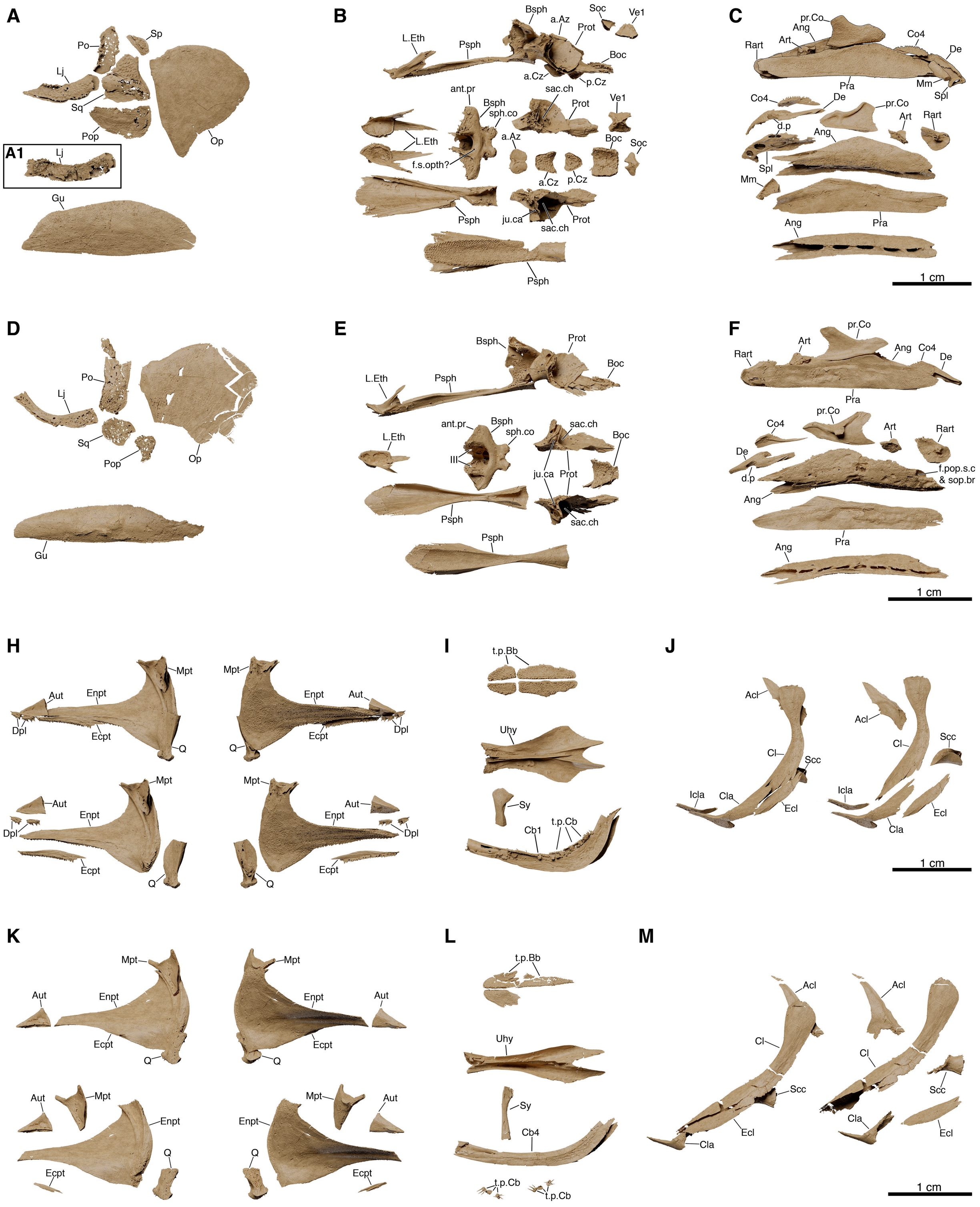
A
Sp
Po
Lj
Sq
Pop
Op
A1
Lj
Gu
B
Bsph
Soc
Ve1
a.Az
Prot
L.Eth
Psph
Boc
a.Cz
p.Cz
ant.pr
sac.ch
Bsph
sph.co
Prot
Ve1
L.Eth
a.Az
Boc
Soc
f.s.opth?
a.Cz
p.Cz
Psph
ju.ca
Prot
sac.ch
Psph
C
pr.Co
Ang
Art
Rart
Co4
De
Pra
Mm
Spl
Co4
De
pr.Co
Art
Rart
d.p
Ang
Spl
Mm
Ang
Pra
1 cm
D
Po
Lj
Sq
Pop
Op
Gu
E
Bsph
Prot
Boc
L.Eth
Psph
ant.pr
Bsph
sph.co
sac.ch
L.Eth
Boc
III
ju.ca
Prot
Psph
sac.ch
Psph
F
pr.Co
Art
Rart
Ang
Co4
De
Pra
pr.Co
Co4
Art
Rart
De
d.p
f.pop.s.c
& sop.br
Ang
Ang
Pra
1 cm
H
Mpt
Aut
Enpt
Dpl
Ecpt
Q
Mpt
Enpt
Aut
Q
Ecpt
Dpl
Mpt
Aut
Enpt
Dpl
Ecpt
Q
Mpt
Aut
Enpt
Dpl
Q
Ecpt
I
t.p.Bb
Uhy
Sy
t.p.Cb
Cb1
J
Acl
Cl
Scc
Icla
Cla
Ecl
Acl
Cl
Scc
Icla
Ecl
Cla
1 cm
K
Mpt
Aut
Enpt
Ecpt
Q
Mpt
Enpt
Aut
Ecpt
Q
Aut
Mpt
Enpt
Q
Ecpt
Mpt
Aut
Enpt
Q
Ecpt
L
t.p.Bb
Uhy
Sy
Cb4
t.p.Cb
t.p.Cb
M
Acl
Acl
Cl
Cl
Scc
Scc
Ecl
Cla
Cla
Ecl
1 cm

Supplementary Fig. 1 (previous page) | Selected skeletal elements of *Graulia branchiodonta* and *Loreleia eucingulata* gen. et sp. nov. based on synchrotron phase-contrast microCT. Elements illustrate morphological differences between the two taxa. The following elements belong to *G. branchiodonta* holotype MHNG-GEPI-V5787: (A) cheek with opercle and gular plate, (B) neurocranium, (C) lower jaw, (H) palatoquadrate, (I) hyobranchial apparatus, and (J) pectoral girdle. The isolated lachrymojugal in (A1) belongs to a referred specimen of *G. branchiodonta* MHNG-GEPI-V5788. The following elements belong to *L. eucingulata* holotype MHNG-GEPI-V5789: (D) cheek with opercle and gular plate, (E) neurocranium, (F) lower jaw, (K) palatoquadrate, (L) hyobranchial apparatus, and (J) pectoral girdle. Abbreviations: Acl, anocleithrum; Ang, angular; Ant.pr, antotic process; Art, articular; Aut, autopalatine; a.Az, anterior anazygal; a.Cz, anterior catazygal; Boc, basioccipital; Bpsh, basisphenoid; Cb4, fourth ceratobranchial; Cl, cleithrum; Co4, fourth coronoid; De, dentary; d.p, dentary pore; Dpl, dermopalatine; Ecl, extracleithrum; Ecpt, ectopterygoid; Enpt, entopterygoid; f.pop.s.c & sop.br, foramen for the preopercular sensory canal and subopercular branch; f.s.opth, foramen for superficial ophthalmic nerve; Gu, gular plate; Icla, interclavicle; III, oculomotor foramen; ju.ca, jugular canal; L.Eth, lateral ethmoid; Lj, lachrymojugal; Mm, mentomeckelian; Mpt, metapterygoid; Op, opercle; p.Cz, posterior catazygal; Po, postorbital; Pop, preopercle; Pra, prearticular; pr.Co, principal coronoid; Prot, prootic; Psph, parasphenoid; Q, quadrate; Rart, retroarticular; sac.ch, saccular chamber; Scc, scapulocoracoid; Sp, spiracular; sph.co, sphenoid condyle; Spl, splenial; spt.fos, suprapterygoid fossa; Sq, squamosal; Sy, symplectic; t.p Bb, tooth plate of basibranchial; t.p Cb, tooth plate of ceratobranchial; Uhy, urohyal; Ve1, first vertebra. A high-resolution version of this figure can be downloaded from Figshare.
(Figshare DOI: https://doi.org/10.6084/m9.figshare.31064614).

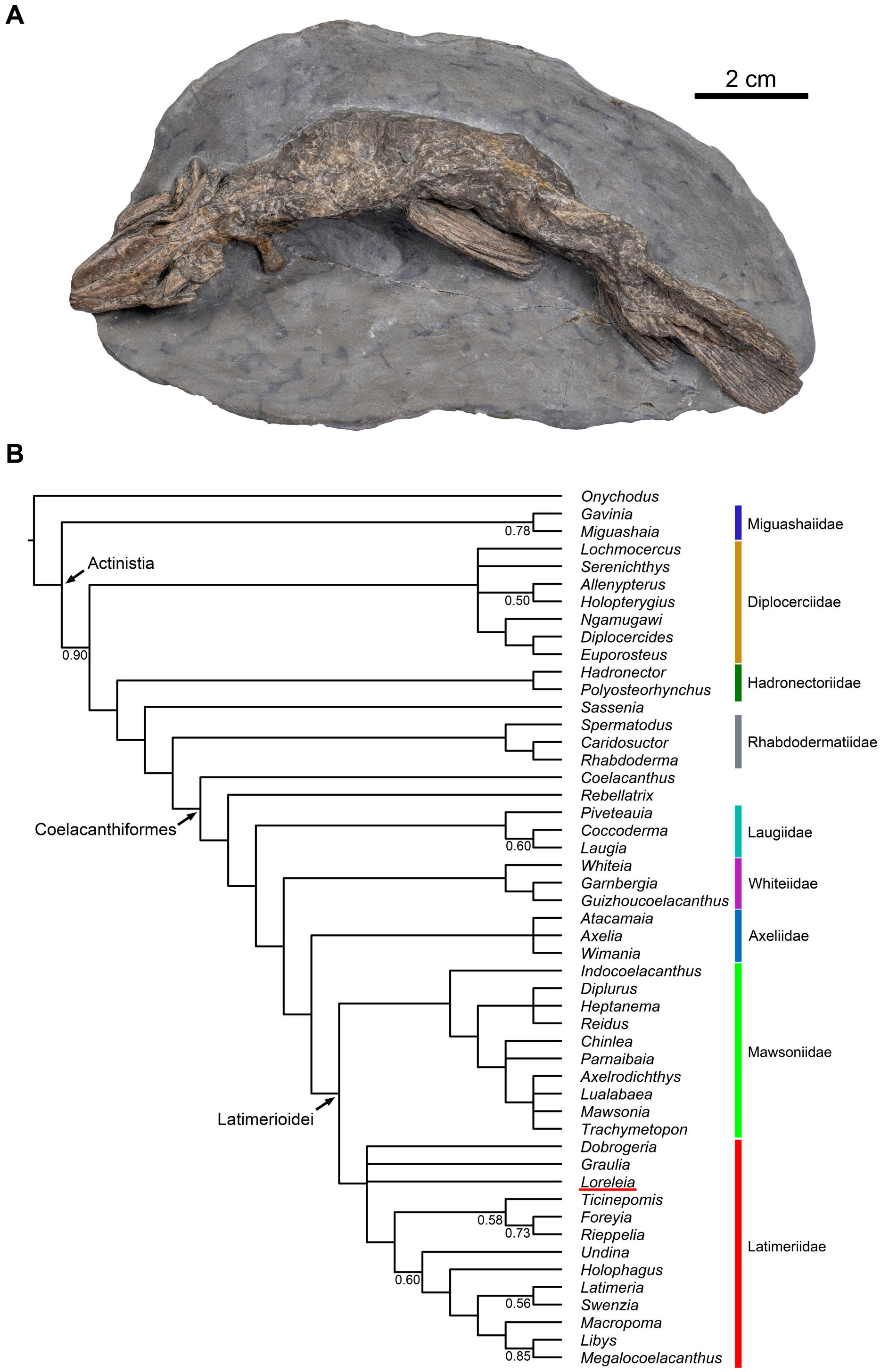

Supplementary Fig. 2 | *Loreleia eucingulata* gen. et sp. nov. (A) Photograph of the holotype MHNG-GEPI-V5789, a nearly complete, three-dimensionally preserved articulated specimen from the Ladinian (Middle Triassic, ~240 Ma) of eastern France. (B) Strict consensus tree from 3,456 equally parsimonious trees (length = 332; consistency index = 0.368; retention index = 0.693), showing the phylogenetic position of *Loreleia* among coelacanth taxa. Bootstrap values >50% are shown. *Loreleia*, *Graulia*, and *Dobrogeria* are recovered as the earliest diverging latimeriids, preceding the split between Latimeriinae and Ticinepomiinae. This portion of coelacanth phylogeny, comprising the most basal mawsoniids and latimeriids, remains unstable, with taxonomic placements varying across studies. A high-resolution version of this figure can be downloaded from Figshare.
(Figshare DOI: https://doi.org/10.6084/m9.figshare.31064614).

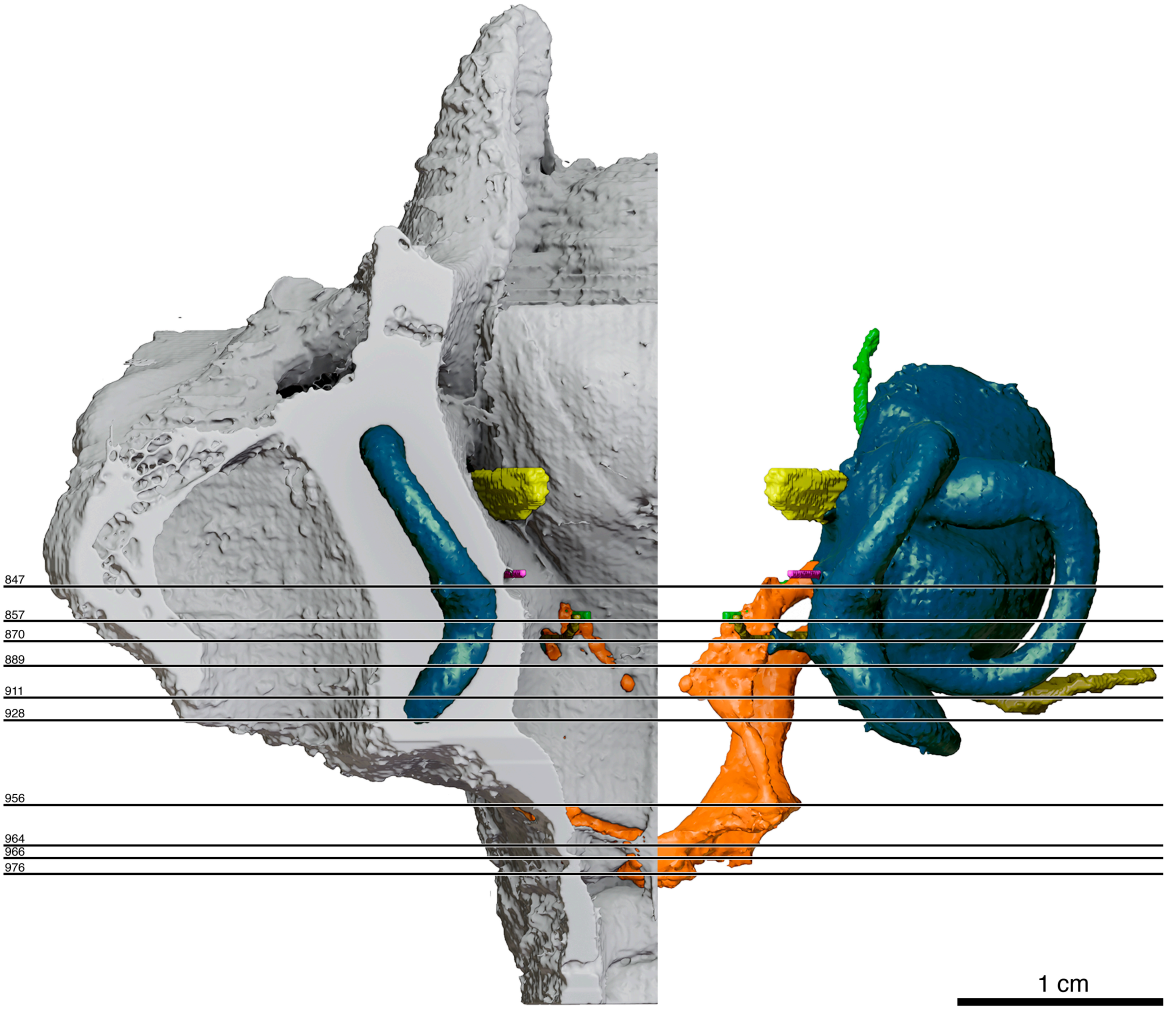

Supplementary Fig. 3 | Otoccipital region of the neurocranium in *Latimeria chalumnae* (Pup2, 356 mm TL, ZSMN-28409, CCC162.21), based on synchrotron phase-contrast microCT. Black lines indicate the positions of histological sections shown in Supplementary Figs. 4–6. Dorsal view showing the inner ear cavity (blue), perilymphatic system (orange), endolymphatic duct (pink), and cranial nerves VIII (yellow), IX (brown), and VI (green). The right half of the neurocranium, skull roof, and brain are omitted. The complex, unpaired perilymphatic system may have connected the inner ear to the lung in extinct coelacanths, potentially enabling sound-pressure detection. A high-resolution version of this figure can be downloaded from Figshare.
(Figshare DOI: https://doi.org/10.6084/m9.figshare.31064614).

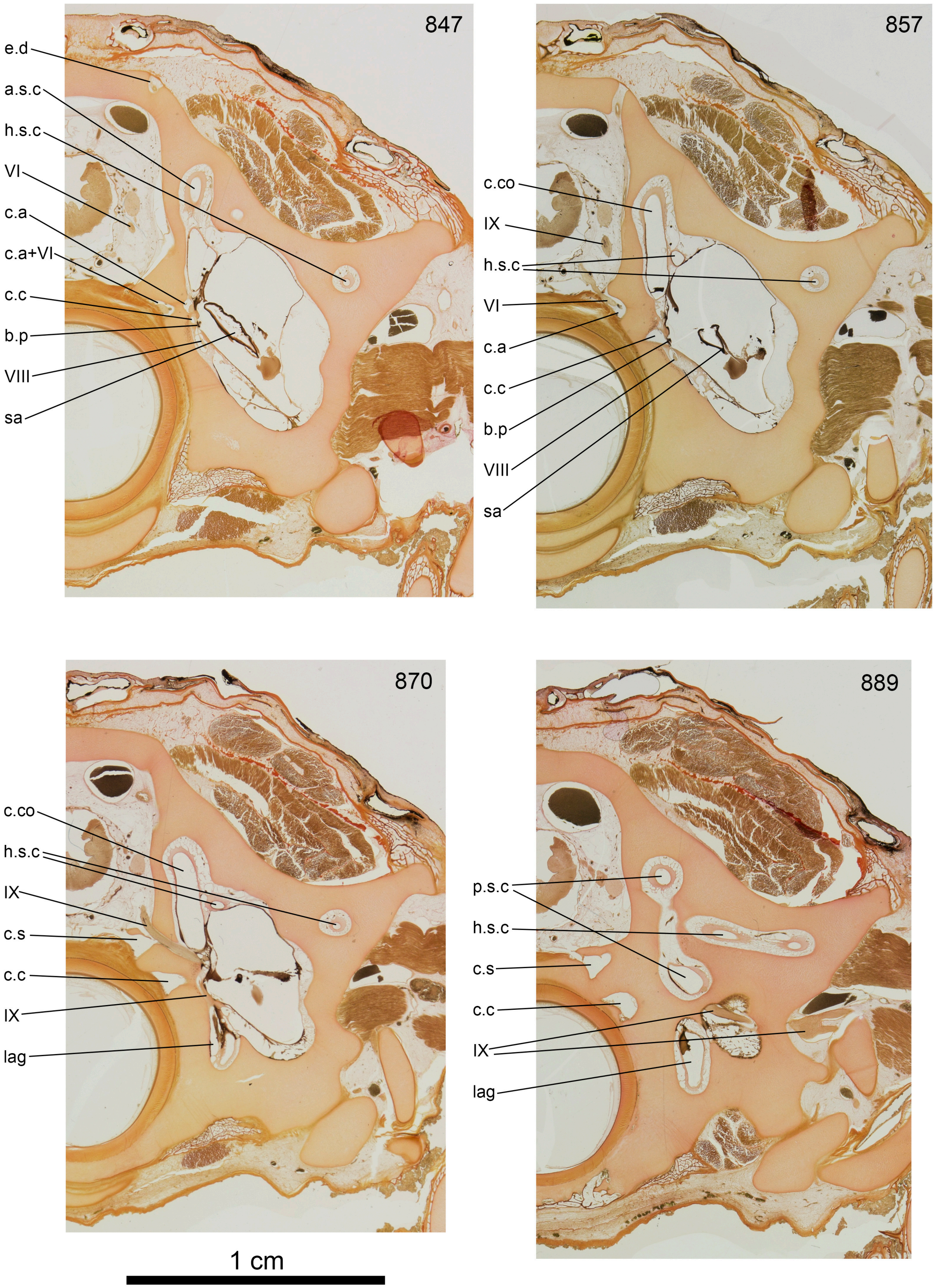

Supplementary Fig. 4 | Archival histological sections of the head of *Latimeria chalumnae* (Pup1b, 303 mm TL, AMNH-32949h, CCC29.1), showing the anterior portion of the canal communicans in contact with the inner ear. Abbreviations: a.s.c, anterior semicircular canal; b.p, basilar papilla; c.a, cochlear aqueduct; c.a+VI, cochlear aqueduct and cranial nerve VI; c.c, canal communicans; c.co, crus communis; c.s, canal superior; e.d, endolymphatic duct; h.s.c, horizontal semicircular canal; IX, glossopharyngeal nerve; lag, lagena; p.s.c, posterior semicircular canal; sa, sagitta; VI, abducens nerve; VIII, vestibulocochlear nerve. A high-resolution version of this figure can be downloaded from Figshare. (Figshare DOI: https://doi.org/10.6084/m9.figshare.31064614).

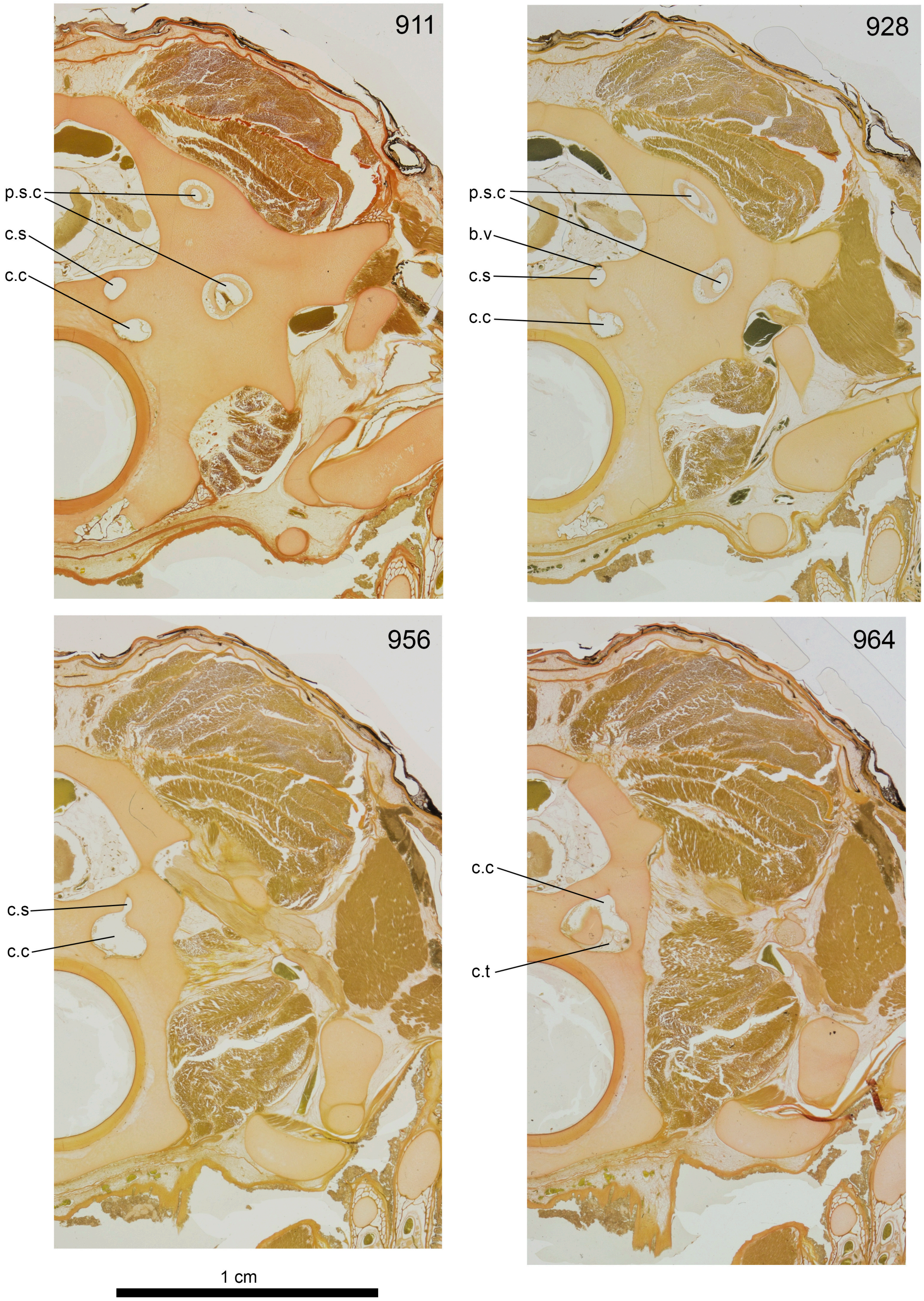

Supplementary Fig. 5 | Archival histological sections of the head of *Latimeria chalumnae* (Pup1b, 303 mm TL, AMNH-32949h, CCC29.1), showing the fusion between the canal superior and the canal communicans. Abbreviations: b.v, blood vessel; c.c, canal communicans; c.s, canal superior; c.t, connective tissue; p.s.c, posterior semicircular canal. A high-resolution version of this figure can be downloaded from Figshare. (Figshare DOI: https://doi.org/10.6084/m9.figshare.31064614).

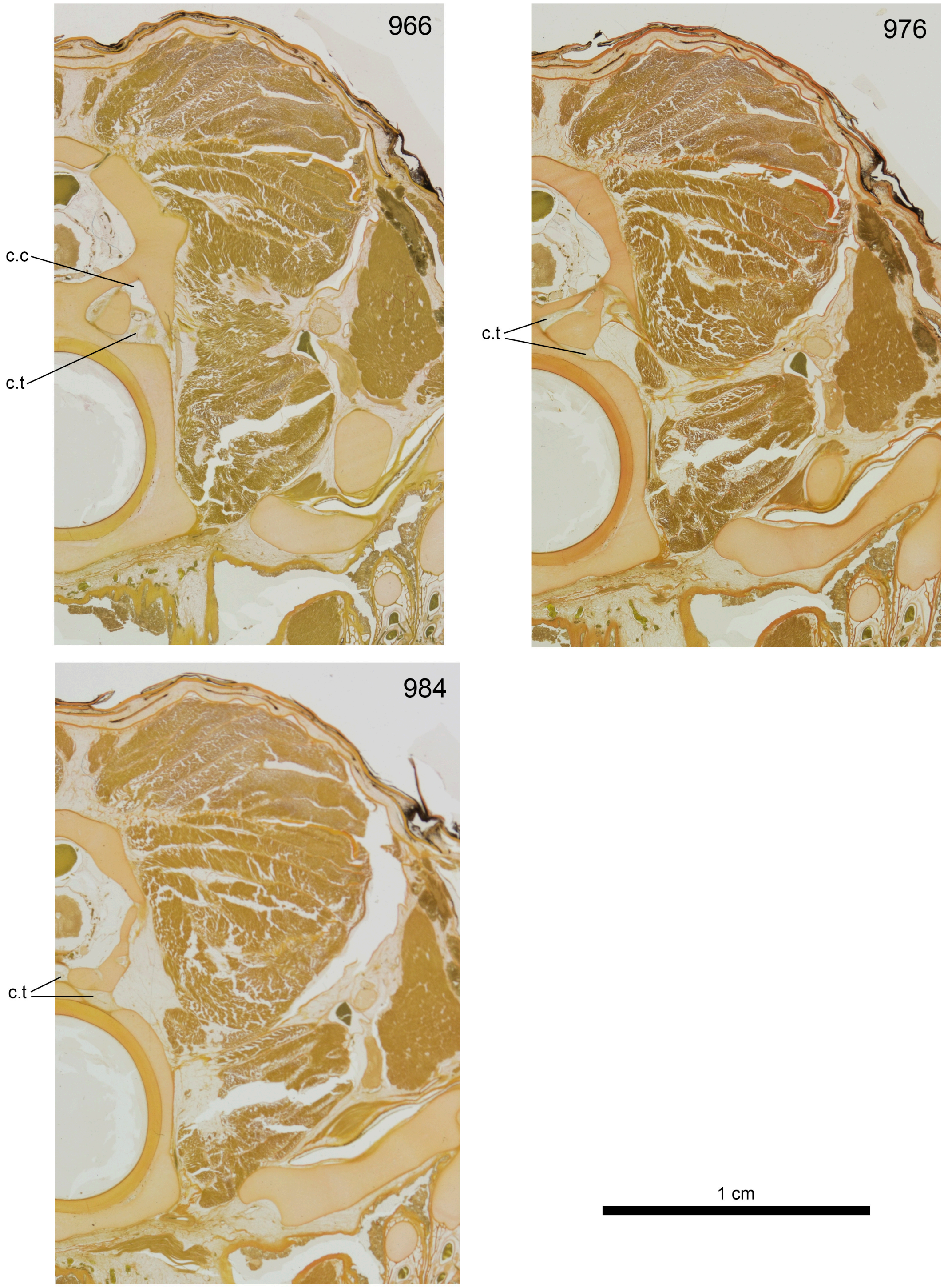

Supplementary Fig. 6 | Archival histological sections of the head of *Latimeria chalumnae* (Pup1b, 303 mm TL, AMNH-32949h, CCC29.1) showing the posterior end of the canal communicans and the commissure filled with connective tissue. Abbreviations – c.c: canalis communicans; c.t: connective tissue. A high-resolution version of this figure can be downloaded from Figshare. (Figshare DOI: https://doi.org/10.6084/m9.figshare.31064614)

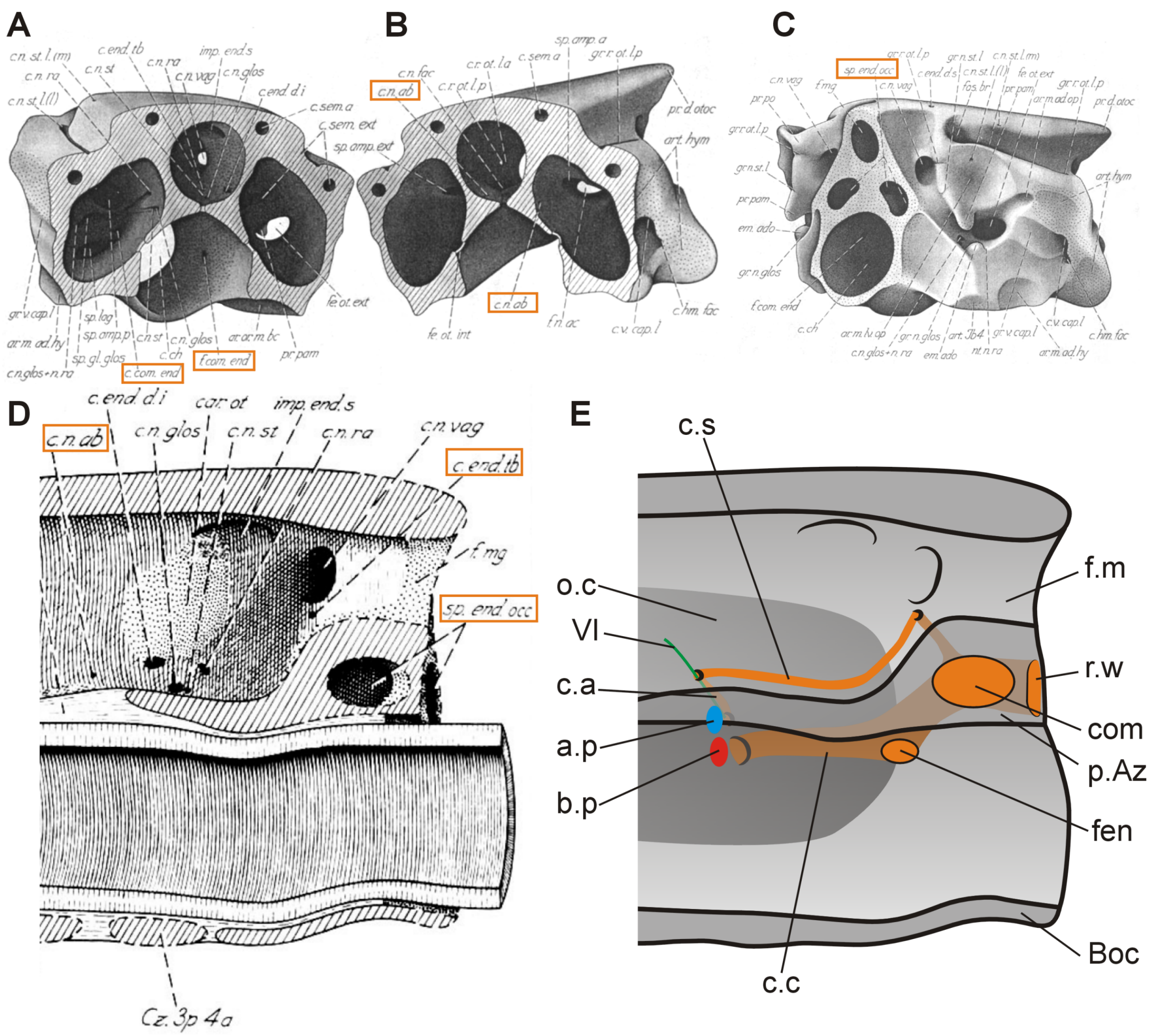

Supplementary Fig. 7 | *Diplocercides kayseri,* reconstruction of the basioccipital portion of the braincase based on a serial grinding and wax plate reconstruction (Stensiö, 1937 and Bjerring, 1972). (A-B) Otico-occipital part bisected crosswise to expose details of its interior (Bjerring, 1972 fig. 2), (C) Posterolateral view of the otico-occipital part (Bjerring, 1972 fig. 4), (D) left view of posterior half of endocranium cut open along the mediosagittal plane (Bjerring, 1972 fig. 1), (E) reconstruction of the perilymphatic system based on (A-D). The abbreviations framed in orange in (A-D) correspond to anatomical structure figured in (E). Abbreviations in (A-D) (from Bjerring, 1972, only the orange-framed ones): c.com.-end, canal for endolymphatic occipital commissure; c.end.tb, canal for tubular branch of occipital commissure between membranous labyrinths; c.n.ab: canal for abducens nerve; f.com.end: foramen between canal for notochord and space for endolymphatic occipital commissure; sp.end.occ: space for endolymphatic occipital commissure. Abbreviations in (E): a.p, amphibian papilla; Boc, basioccipital; b.p, basilar papilla (supposed location); c.a, cochlear aqueduct; c.c, canal communicans; com, commissure of the canal communicans; c.s, canal superior; fen, fenestra between canal for notochord and canal communicans; f.m, foramen magnum; o.c, otic capsule; p.Az, posterior anazygal; r.w, round window; VI, abducens nerve. A high-resolution version of this figure can be downloaded from Figshare (Figshare DOI: https://doi.org/10.6084/m9.figshare.31064614)
