# Supplementary material for: A dual respiratory and auditory function for the coelacanth lung
Source: Commun Biol. 2026 Feb 14;9:400. doi: 10.1038/s42003-026-09708-6 (PMC13003144; doi:10.1038/s42003-026-09708-6)
Supplement: Supplementary file 2 — Description of Additional Supplementary File [file 42003_2026_9708_MOESM2_ESM.pdf]

Description of Additional Supplementary Files

File name: Supplementary Data 1

Description: Data matrix of coelacanths employed in the phylogenetic analysis and list of characters.

File name: Supplementary Movie 1

Description: 360° rotation of the reconstructed skull and anterior lung chamber of Graulia branchiodonta holotype MHNG-GEPIV5787.

File name: Supplementary Movie 2

Description: 360° rotation of the reconstructed skull and anterior lung chamber of Loreleia eucingulata holotype MHNG-GEPI-V5789.

File name: Supplementary Movie 3

Description: 360° rotation of the otoccipital neurocranium of Latimeria chalumnae pup2, 356 mm TL, ZSMN-28409, CCC162.21, showing the inner ear cavity (blue), perilymphatic system (orange), endolymphatic duct (pink) and nerves VIII (yellow), IX (brown), VI (green). The skull roof has been removed; the brain is not shown.

File name: Supplementary Movie 4

Description: 360° rotation of the isolated left inner ear of Latimeria chalumnae pup1a, 305 mm TL, MNHN-AC-2012-22, CCC29.5, showing the inner ear cavity (blue), perilymphatic system (orange), endolymphatic duct (pink) and nerves VIII (yellow), IX (brown), VI (green). Progressive dissection during the video reveals the saccular (blue) and utricular (purple) membranes and the otoliths (grey).
