# Supplementary material for: A dual respiratory and auditory function for the coelacanth lung
Source: Commun Biol. 2026 Feb 14;9:400. doi: 10.1038/s42003-026-09708-6 (PMC13003144; doi:10.1038/s42003-026-09708-6)
Supplement: Supplementary file 8 — Reporting-summary [file 42003_2026_9708_MOESM8_ESM.pdf]

Corresponding author(s): Luigi Manuelli

Last updated by author(s): Jan 20, 2026

Reporting Summary

Nature Portfolio wishes to improve the reproducibility of the work that we publish. This form provides structure for consistency and transparency in reporting. For further information on Nature Portfolio policies, see our Editorial Policies and the Editorial Policy Checklist.

Statistics

For all statistical analyses, confirm that the following items are present in the figure legend, table legend, main text, or Methods section.

n/a	Confirmed	
☐	☒	The exact sample size (*n*) for each experimental group/condition, given as a discrete number and unit of measurement
☐	☒	A statement on whether measurements were taken from distinct samples or whether the same sample was measured repeatedly
☒	☐	The statistical test(s) used AND whether they are one- or two-sided<br>*Only common tests should be described solely by name; describe more complex techniques in the Methods section.*
☒	☐	A description of all covariates tested
☒	☐	A description of any assumptions or corrections, such as tests of normality and adjustment for multiple comparisons
☒	☐	A full description of the statistical parameters including central tendency (e.g. means) or other basic estimates (e.g. regression coefficient) AND variation (e.g. standard deviation) or associated estimates of uncertainty (e.g. confidence intervals)
☒	☐	For null hypothesis testing, the test statistic (e.g. *F*, *t*, *r*) with confidence intervals, effect sizes, degrees of freedom and *P* value noted<br>*Give P values as exact values whenever suitable.*
☒	☐	For Bayesian analysis, information on the choice of priors and Markov chain Monte Carlo settings
☒	☐	For hierarchical and complex designs, identification of the appropriate level for tests and full reporting of outcomes
☒	☐	Estimates of effect sizes (e.g. Cohen's *d*, Pearson's *r*), indicating how they were calculated

Our web collection on statistics for biologists contains articles on many of the points above.

Software and code

Policy information about availability of computer code

Data collection	Data were acquired using Propagation Phase Contrast Synchrotron Radiation micro-Computed Tomography (PPC-SRμCT) at ESRF (beamlines BM05 and ID19) and conventional CT (Siemens Somatom Definition Dual Energy CT). Raw imaging data were collected using detector-specific acquisition software provided by the ESRF and CT manufacturer. No custom code was used for initial data acquisition.
Data analysis	3D segmentation and rendering of anatomical structures were performed using: Dragonfly 4.1 (Comet) – for manual segmentation and local thresholding of CT volumes. Blender 3.1.2 – for reconstruction, repositioning of skeletal elements, and rendering of 3D models. Adobe Photoshop 2021 – for image refinement and figure plate assembly. Phylogenetic analyses were performed using PAUP 4.0* with heuristic searches, TBR branch swapping, and bootstrap assessment. Custom scripts were not used; all analyses were performed using standard software features.

For manuscripts utilizing custom algorithms or software that are central to the research but not yet described in published literature, software must be made available to editors and reviewers. We strongly encourage code deposition in a community repository (e.g. GitHub). See the Nature Portfolio guidelines for submitting code & software for further information.

Data

Policy information about availability of data

All manuscripts must include a data availability statement. This statement should provide the following information, where applicable:

- Accession codes, unique identifiers, or web links for publicly available datasets
- A description of any restrictions on data availability
- For clinical datasets or third party data, please ensure that the statement adheres to our policy

The synchrotron scan files of Graulia branchiodonta (MHNG-GEPI-V5787, holotype and MHNG-GEPI-V5787, referred specimen) are available from the ESRF Paleontology Database (https://paleo.esrf.fr/datasets/2015882168 ; https://paleo.esrf.fr/datasets/2015882170)41,42. The synchrotron scan files of Loreleia eucingulata (MHNG-GEPI-V5789, holotype) will be publicly available from the ESRF Paleontology Database (https://paleo.esrf.fr/) along with surface files of the individual bones. The fossil material housed in the Natural History Museum of Geneva is available for study upon request.

Research involving human participants, their data, or biological material

Policy information about studies with human participants or human data. See also policy information about sex, gender (identity/presentation), and sexual orientation and race, ethnicity and racism.

Reporting on sex and gender	This study did not involve human participants, and no human data were collected. Therefore, sex and gender were not relevant to the study design or analyses, and no sex- or gender-based analyses were performed.
Reporting on race, ethnicity, or other socially relevant groupings	No socially constructed or socially relevant human groupings were used, as the study did not involve human participants.
Population characteristics	The study exclusively analyzed fossil specimens and archived material of extant coelacanths; no human participants were recruited or included.
Recruitment	The study exclusively analyzed fossil specimens and archived material of extant coelacanths; no human participants were recruited or included.
Ethics oversight	No human research ethics approval was required because no human participants or human biological materials were used.

Note that full information on the approval of the study protocol must also be provided in the manuscript.

Field-specific reporting

Please select the one below that is the best fit for your research. If you are not sure, read the appropriate sections before making your selection.

☐ Life sciences ☐ Behavioural & social sciences ☒ Ecological, evolutionary & environmental sciences

For a reference copy of the document with all sections, see nature.com/documents/nr-reporting-summary-flat.pdf

Ecological, evolutionary & environmental sciences study design

All studies must disclose on these points even when the disclosure is negative.

Study description	This study investigated the anatomy and potential function of the lung and inner ear in both fossil and extant coelacanths. It combined synchrotron phase-contrast microCT (PPC-SRµCT) imaging of two 240-million-year-old fossil latimerioid coelacanths (Graulia branchiodonta and Loreleia eucingulata), with multiple developmental stages of the extant Latimeria chalumnae. The study applied 3D reconstruction and segmentation to explore lung and perilymphatic system morphology. No experimental treatments were applied; the design is observational and comparative across species and ontogenetic stages.
Research sample	Fossil specimens - Graulia branchiodonta: holotype (MHNG-GEPI-V5787) and referred specimen (MHNG-GEPI-V5788). Loreleia eucingulata: holotype (MHNG-GEPI-V5789). Extant specimens - Latimeria chalumnae: Pup1a (MNHN-AC-2012-22), Pup1b (AMNH-32949h), Pup2 (ZSMN-28409), Adult male: ZMUC-P1112. The fossil samples represent Triassic latimerioid coelacanths from eastern France, selected to study lung morphology. Extant specimens cover juvenile and adult stages of L. chalumnae. Juvenile specimens were chosen to study inner ear morphology during development; the adult provided comparative anatomical context.
Sampling strategy	Specimens were selected based on completeness and preservation. Fossil specimens were all known representatives of their genera; extant specimens were chosen to include developmental variation. No statistical sample-size calculation was performed due to the rarity of fossil material and extant specimens. Sample sizes were sufficient to observe and reconstruct the lung and perilymphatic anatomy, fulfilling the study's comparative anatomical objectives.
Data collection	Data were acquired via PPC-SRµCT at ESRF (beamlines BM05 and ID19) for fossils (in 2022 by Luigi Manuelli, Lionel Cavin, Kathleen Dollmann, Kudakwashe Jakata) and extant pups (by Hugo Dutel and Paul Tafforeau, data publicly released in 2024), and conventional CT for the adult L. chalumnae (by Henrik Lauridsen, data publicly released in 2021). Archival histological sections of Pup1b were

digitized and provided by the American Museum of Natural History. Segmentation and 3D reconstructions were performed manually in Dragonfly 4.1 and Blender 3.1.2 by Luigi Manuelli, guided by anatomical reference models.

Timing and spatial scale	Imaging was conducted during scheduled synchrotron beamline access.
Data exclusions	No data were excluded from analyses.
Reproducibility	Reproducibility was ensured through independent imaging of multiple specimens and repeated 3D reconstruction of perilymphatic structures (for L. chalumnae, two pups, adult, and histological sections) and fossilized lung structures (two specimens of G. branchiodonta and one specimen of L. eugcingulata). All reconstruction attempts were successful and consistent across specimens.
Randomization	Randomization was not applicable because the study was observational and comparative; no experimental grouping was used.
Blinding	Blinding was not applicable. Data acquisition and analysis required detailed anatomical expertise and direct interaction with specimens, which precluded blinding. However, segmentation and reconstruction were conducted systematically following established anatomical criteria.

Did the study involve field work? ☐ Yes ☒ No

Reporting for specific materials, systems and methods

We require information from authors about some types of materials, experimental systems and methods used in many studies. Here, indicate whether each material, system or method listed is relevant to your study. If you are not sure if a list item applies to your research, read the appropriate section before selecting a response.

Materials & experimental systems

n/a	Involved in the study	
☒	☐	Antibodies
☒	☐	Eukaryotic cell lines
☐	☒	Palaeontology and archaeology
☐	☒	Animals and other organisms
☒	☐	Clinical data
☒	☐	Dual use research of concern
☒	☐	Plants

Methods

n/a	Involved in the study	
☒	☐	ChIP-seq
☒	☐	Flow cytometry
☒	☐	MRI-based neuroimaging

Palaeontology and Archaeology

Specimen provenance	Fossil specimens were collected from eastern France. Graulia branchiodonta: MHNG-GEPI-V5787 (holotype) and MHNG-GEPI-V5788 (referred specimen), Ladinian (Middle Triassic) deposits, Sarraltroff, Moselle, Grand Est, France, Calcaire à Cératites Formation, Ceratites praenodosus biozone. Loreleia eucingulata: MHNG-GEPI-V5789 (holotype), same locality and horizon as G. branchiodonta. Extant specimens of Latimeria chalumnae were obtained from historical collections housed in: MNHN (France), AMNH (USA), ZSMN (Germany), and ZMUC (Denmark). The acquisition of extant specimens adhered to institutional collection policies; no new collection of live animals was performed. Permits for fossil collection were issued by local authorities in France at the time of excavation; specific issuing authority and date are not reported in the manuscript.
Specimen deposition	All fossil specimens are deposited at the Natural History Museum of Geneva (MHNG). Extant coelacanth specimens are housed in their respective institutions: MNHN, AMNH, ZSMN, ZMUC. These repositories allow access for study by other researchers. Synchrotron scan files for Graulia branchiodonta are available via the ESRF Paleontology Database; scans for Loreleia eucingulata will be made publicly available upon publication.
Dating methods	No new radiometric or geochronological dates were generated for this study. Fossil specimens are dated based on stratigraphic correlation to the Middle Triassic Muschelkalk, Calcaire à Cératites Formation, Ceratites praenodosus biozone (Ladinian, 242–237 Mya).

☒ Tick this box to confirm that the raw and calibrated dates are available in the paper or in Supplementary Information.

Ethics oversight	No ethical approval was required for this study because the work involved fossil specimens and historical museum material. Extant specimens were studied from archived museum collections; no live animals were collected or manipulated.

Note that full information on the approval of the study protocol must also be provided in the manuscript.

Animals and other research organisms

Policy information about studies involving animals; ARRIVE guidelines recommended for reporting animal research, and Sex and Gender in Research

Laboratory animals	This study did not involve laboratory animals.
Wild animals	No wild animals were observed, captured, or manipulated in the field. The study used only fossil specimens and archived museum specimens of the extant Latimeria chalumnae.
Reporting on sex	Sex was recorded for extant Latimeria chalumnae specimens when known: Pup1a (MNHN-AC-2012-22): sex not determined. Pup1b (AMNH-32949h): sex not determined. Pup2 (ZSMN-28409): sex not determined. Adult (ZMUC-P1112): male. No sex-based analyses were performed because the study focused on comparative anatomy of lung and inner ear structures across developmental stages and fossil taxa; sex was not expected to affect the morphological traits of interest.
Field-collected samples	Not applicable. All extant specimens were studied from archived museum collections; no new field-collected animals were used.
Ethics oversight	No animal research ethics approval was required because no live animals were collected, housed, or experimentally manipulated. All extant specimens were obtained from historical collections and studied post-mortem.

Note that full information on the approval of the study protocol must also be provided in the manuscript.

Plants

Seed stocks	No plants or plant-derived material were used in this study.
Novel plant genotypes	No plants or plant-derived material were used in this study.
Authentication	No plants or plant-derived material were used in this study.
